# Standard chemotherapy with or without bevacizumab for women with newly diagnosed ovarian cancer (ICON7): overall survival results of a phase 3 randomised trial

**DOI:** 10.1016/S1470-2045(15)00086-8

**Published:** 2015-08

**Authors:** Amit M Oza, Adrian D Cook, Jacobus Pfisterer, Andrew Embleton, Jonathan A Ledermann, Eric Pujade-Lauraine, Gunnar Kristensen, Mark S Carey, Philip Beale, Andrés Cervantes, Tjoung-Won Park-Simon, Gordon Rustin, Florence Joly, Mansoor R Mirza, Marie Plante, Michael Quinn, Andrés Poveda, Gordon C Jayson, Dan Stark, Ann Marie Swart, Laura Farrelly, Richard Kaplan, Mahesh K B Parmar, Timothy J Perren

**Affiliations:** a Princess Margaret Cancer Centre, Toronto, ON, Canada; b Medical Research Council Clinical Trials Unit at University College London, London, UK; c Gynecologic Oncology Center, AGO Study Group, Kiel, Germany; d Cancer Clinical Trials Unit, University College London Hospital, London, UK; e AP-HP, Site Hôtel-Dieu, Université Paris Descartes, Paris, France; f Department of Gynaecologic Oncology and Institute for Cancer Genetics and Informatics, Oslo University Hospital, Institute for Clinical Medicine, Oslo University, Norway; g University of British Columbia, Vancouver, Canada; h Concord Hospital, Sydney, NSW, Australia; i Department of Haematology and Medical Oncology, Biomedical Research Institute INCLIVA, University of Valencia, Valencia, Spain; j Department of Obstetrics and Gynecology, Hannover Medical School, Hannover, Germany; k Mount Vernon Hospital, London, UK; l Centre François Baclesse, Caen, France; m Department of Oncology, Copenhagen University Hospital, Copenhagen, Denmark; n Centre Hospitalier Universitaire de Québec, L'Hôtel-Dieu de Québec, Canada; o Royal Women's Hospital, Melbourne, Australia; p Fundación Instituto Valenciano de Oncología, Valencia, Spain; q Institute of Cancer Sciences and Christie Hospital, University of Manchester, Manchester, UK; r St James Institute of Oncology, St James University Hospital, Leeds, UK; s Norwich Clinical Trials Unit, University of East Anglia, Norwich, UK

## Abstract

**Background:**

The ICON7 trial previously reported improved progression-free survival in women with ovarian cancer with the addition of bevacizumab to standard chemotherapy, with the greatest effect in patients at high risk of disease progression. We report the final overall survival results of the trial.

**Methods:**

ICON7 was an international, phase 3, open-label, randomised trial undertaken at 263 centres in 11 countries across Europe, Canada, Australia and New Zealand. Eligible adult women with newly diagnosed ovarian cancer that was either high-risk early-stage disease (International Federation of Gynecology and Obstetrics [FIGO] stage I–IIa, grade 3 or clear cell histology) or more advanced disease (FIGO stage IIb–IV), with an Eastern Cooperative Oncology Group performance status of 0–2, were enrolled and randomly assigned in a 1:1 ratio to standard chemotherapy (six 3-weekly cycles of intravenous carboplatin [AUC 5 or 6] and paclitaxel 175 mg/m^2^ of body surface area) or the same chemotherapy regimen plus bevacizumab 7·5 mg per kg bodyweight intravenously every 3 weeks, given concurrently and continued with up to 12 further 3-weekly cycles of maintenance therapy. Randomisation was done by a minimisation algorithm stratified by FIGO stage, residual disease, interval between surgery and chemotherapy, and Gynecologic Cancer InterGroup group. The primary endpoint was progression-free survival; the study was also powered to detect a difference in overall survival. Analysis was by intention to treat. This trial is registered as an International Standard Randomised Controlled Trial, number ISRCTN91273375.

**Findings:**

Between Dec 18, 2006, and Feb 16, 2009, 1528 women were enrolled and randomly assigned to receive chemotherapy (n=764) or chemotherapy plus bevacizumab (n=764). Median follow-up at the end of the trial on March 31, 2013, was 48·9 months (IQR 26·6–56·2), at which point 714 patients had died (352 in the chemotherapy group and 362 in the bevacizumab group). Our results showed evidence of non-proportional hazards, so we used the difference in restricted mean survival time as the primary estimate of effect. No overall survival benefit of bevacizumab was recorded (restricted mean survival time 44·6 months [95% CI 43·2–45·9] in the standard chemotherapy group *vs* 45·5 months [44·2–46·7] in the bevacizumab group; log-rank p=0·85). In an exploratory analysis of a predefined subgroup of 502 patients with poor prognosis disease, 332 (66%) died (174 in the standard chemotherapy group and 158 in the bevacizumab group), and a significant difference in overall survival was noted between women who received bevacizumab plus chemotherapy and those who received chemotherapy alone (restricted mean survival time 34·5 months [95% CI 32·0–37·0] with standard chemotherapy *vs* 39·3 months [37·0–41·7] with bevacizumab; log-rank p=0·03). However, in non-high-risk patients, the restricted mean survival time did not differ significantly between the two treatment groups (49·7 months [95% CI 48·3–51·1]) in the standard chemotherapy group *vs* 48·4 months [47·0–49·9] in the bevacizumab group; p=0·20). An updated analysis of progression-free survival showed no difference between treatment groups. During extended follow-up, one further treatment-related grade 3 event (gastrointestinal fistula in a bevacizumab-treated patient), three grade 2 treatment-related events (cardiac failure, sarcoidosis, and foot fracture, all in bevacizumab-treated patients), and one grade 1 treatment-related event (vaginal haemorrhage, in a patient treated with standard chemotherapy) were reported.

**Interpretation:**

Bevacizumab, added to platinum-based chemotherapy, did not increase overall survival in the study population as a whole. However, an overall survival benefit was recorded in poor-prognosis patients, which is concordant with the progression-free survival results from ICON7 and GOG-218, and provides further evidence towards the optimum use of bevacizumab in the treatment of ovarian cancer.

**Funding:**

The National Institute for Health Research through the UK National Cancer Research Network, the Medical Research Council, and Roche.

Panel **Research in context**

**Evidence before this study**

The primary progression-free survival analysis of the ICON7 trial reported significantly improved progression-free survival when bevacizumab was added to standard chemotherapy in newly diagnosed ovarian cancer. The effect was greatest in patients at high risk of disease progression. Similar progression-free survival findings were reported in the GOG-218 trial.

**Added value of this study**

In a planned mature analysis of overall survival, no difference in overall survival was noted between those patients who received bevacizumab plus chemotherapy and those who received chemotherapy alone. However, in subgroup analyses, improved overall survival was noted in patients at high risk of disease progression who received bevacizumab compared with those who did not.

**Implications of all the available evidence**

Bevacizumab may have a role in the treatment of patients with poor-prognosis ovarian cancer. Future work should address questions of treatment duration, targeting, timing, re-challenge, and dose fractionation.

## Introduction

Ovarian cancer is the seventh most common cancer worldwide, with 238 700 new cases and 151 900 deaths in 2012.^1^ The prognosis of the disease remains poor: the European mean age-standardised 5-year survival was only 37·6% for women diagnosed between 2000 and 2007.^2^

Until 2011, the international standard of care for women with advanced or poor-prognosis early-stage ovarian cancer mainly consisted of debulking surgery followed by chemotherapy with carboplatin and paclitaxel.^3^ Since then, modulation of VEGF has moved from a theoretical concept^4^ to a key component of treatment. Two large-scale phase 3 randomised trials, GOG-218 and ICON7, both undertaken in first-line settings, showed that the addition of the anti-VEGF monoclonal antibody, bevacizumab, to conventionally administered carboplatin and paclitaxel chemotherapy significantly improved progression-free survival.^5^, ^6^ Two further randomised trials in recurrent ovarian cancer have shown significant improvement in progression-free survival through the addition of bevacizumab to conventionally administered carboplatin and gemcitabine chemotherapy both in the platinum-sensitive and platinum-resistant relapsed setting.^7^, ^8^ These data were used to support bevacizumab licensing through the European Medicines Agency for use in the first-line setting for patients with at least International Federation of Gynecology and Obstetrics (FIGO) stage IIIB disease (according to 1988 staging criteria), at first recurrence for patients with platinum-sensitive disease not previously treated with bevacizumab or other VEGF-targeted drugs, and in the setting of platinum-resistant recurrence combined with paclitaxel, topotecan, or pegylated liposomal doxorubicin.^9^ Bevacizumab has also been approved in the platinum-refractory setting by the US Food and Drug Administration.^10^

The GOG-218 trial,^5^ which enrolled 1873 patients with FIGO stage III–IV ovarian cancer with macroscopic residual disease after primary surgery, showed a significant improvement in progression-free survival with the addition of bevacizumab (hazard ratio [HR] 0·72, 95% CI 0·63–0·82; p<0·001). Patients received treatment with carboplatin and paclitaxel chemotherapy, and either concurrent bevacizumab 15 mg per kg every 3 weeks followed by up to 16 cycles of maintenance bevacizumab (at the same dose) or placebo for the same duration, all administered intravenously. The ICON7 trial,^6^ which was done in a patient population that included those with poor-prognosis, early-stage disease and those with optimally or suboptimally debulked advanced disease, showed improved progression-free survival in patients receiving bevacizumab, with restricted mean progression-free survival over 36 months of 20·3 months on standard chemotherapy and 21·8 months with bevacizumab (HR 0·81, 95% CI 0·70–0·94; p=0·004). An increased effect was noted in patients at high risk of disease progression, a similar group to the GOG-218 study population, with restricted mean progression-free survival after 42 months' follow-up of 14·5 months in the standard chemotherapy group and 18·1 months in the bevacizumab group (HR 0·73, 95% CI 0·60–0·93; p=0·002).

To fully assess a new treatment, the effect on overall survival as well as progression-free survival must be known. ICON7 was designed with a progression-free survival primary endpoint but was also powered to detect an overall survival improvement. At the time of the primary progression-free survival analysis, overall survival data were immature in both ICON7 and GOG-218, and preliminary analyses showed no difference between treatment groups in either study. Here, we present the final analysis of mature overall survival data from ICON7, together with detailed data about the effect of bevacizumab according to stage and extent of residual disease after primary debulking surgery.

## Methods

### Study design and participants

The design of this trial has previously been described in detail.^6^ In brief, eligible women with ovarian cancer were recruited from 263 centres (a mixture of general hospitals and specialist centres) in 11 countries across Europe, Canada, Australia, and New Zealand (appendix pp 1–5) and were randomly assigned 1:1 in an open-label study to receive standard chemotherapy or standard chemotherapy with bevacizumab. Eligible patients were aged 18 years or older; with newly diagnosed epithelial ovarian, fallopian tube, or primary peritoneal cancer; an Eastern Cooperative Oncology Group (ECOG) performance status of 0–2; FIGO 1988 stage IIb–IV or high-risk (grade 3 or clear cell histology) stage I–IIa disease; had undergone debulking cytoreductive surgery or, in advanced disease, had a biopsy with no further surgery planned; and had adequate coagulation parameters and liver, renal, and bone marrow function. The exclusion criteria were having other tumour types, previous systemic therapy, planned surgery, and uncontrolled hypertension.

The study protocol was compliant with good clinical practice guidelines and the Declaration of Helsinki. Ethics approval was obtained in all participating countries and where required in all participating centres. All patients provided written informed consent.

### Randomisation and masking

Randomisation was done centrally by a computer system based at the Medical Research Council Clinical Trials Unit (London, UK) accessed via the web or telephone. Randomisation was done using 1:1 allocation and a minimisation algorithm, and was stratified by Gynecologic Cancer Intergroup (GCIG) group, a combination of FIGO stage and residual disease (stage I–III and ≤1 cm residual disease *vs* stage I–III and >1 cm residual disease *vs* inoperable stage III and stage IV disease) and planned interval between surgery and chemotherapy (≤4 weeks or >4 weeks). Neither patients nor physicians were masked to treatment allocation.

### Procedures

Patients received either six 3-weekly cycles of intravenous carboplatin (AUC 5 or 6) and paclitaxel (175 mg/m^2^ of body surface area), or the same regimen with intravenous bevacizumab (7·5 mg/kg of bodyweight) given concurrently and continued for 12 further 3-weekly cycles (with a duration of bevacizumab exposure of about 1 year), or until disease progression. To avoid delayed wound healing, bevacizumab was omitted at cycle 1 if chemotherapy was started within 4 weeks of surgery. Bevacizumab cycles omitted for any reason were not replaced.

CT scans were done after treatment cycles 3 and 6, and then 9 and 12 months after randomisation. Following treatment, women were seen every 3 months until the end of year 3, every 6 months during years 4 and 5, and annually thereafter. Scans continued every 6 months until the end of year 3, then as clinically indicated. Disease progression was assessed by investigators according to RECIST 2000^11^ guidelines, and needed radiological or clinical evidence of progression. Cancer antigen 125 (CA125) progression alone was insufficient to define progressive disease. Following disease progression, women were seen every 6 months up until year 5, then annually. Quality of life was assessed with the European Organisation for Research and Treatment of Cancer QLQ-C30 and QLQ-OV28 questionnaires.

### Outcomes

The primary outcome of ICON7 was progression-free survival, which has been previously reported.^6^ Secondary outcomes were overall survival and safety outcomes of adverse events, laboratory results, and worsened ECOG performance status. Exploratory outcome measures were quality of life, health economics, and translational research.

Risk groups were defined at the time of the primary progression-free survival analysis to enable comparison with the GOG-218 study population. They were refined slightly before database lock for the present analysis in accordance with current clinical practice and defined prospectively in the statistical analysis plan. High risk of progression was defined as stage IV disease, inoperable stage III disease, or suboptimally debulked (>1 cm) stage III disease (appendix p 10). To enable comparison with previous analyses, results are also presented for two other high-risk definitions: exclusion of inoperable stage III–IV patients, to match the previous ICON7 high-risk group; and inclusion of patients with 0–1 cm residual tumour, to match the GOG-218 population (appendix p 8). Between the primary progression-free survival analysis and the present analysis, recruiting centres were asked (following the GCIG fourth ovarian cancer consensus conference statement^3^) to reclassify patients with up to 1 cm of residual disease into those with no macroscopic residuum or those with macroscopic residuum measuring 1 cm or less. The non-high-risk patients were defined as those who did not meet the criteria for high-risk disease.

Three further patient subgroups of particular interest were defined: patients with clear cell carcinoma (roughly 10% of patients because of enriched enrolment—ie, the use of less restrictive staging criteria, which meant that patients with clear cell carcinoma of any stage were eligible); high-risk low-stage patients, with stage I–IIA clear cell or grade 3 carcinoma; and low-grade serous carcinomas (grade 1).

### Statistical analysis

This study was designed and powered to detect differences in progression-free and overall survival between the treatment groups. The analysis of overall survival needed 715 deaths to detect a 10-month improvement in median survival from 43 to 53 months (HR 0·81), with 80% power at a two-sided 5% significance level. The progression-free survival analysis needed 684 disease progression events to show a 5-month progression-free survival increase from 18 to 23 months, with 90% power and two-sided 5% significance level.

Analysis followed the principle of intention-to-treat and included all patients randomly assigned to treatment. The primary analysis used an unstratified log-rank test to compare overall survival between randomised groups. Treatment effects were estimated from Cox regression analyses when proportional hazards could be assumed. With evidence of non-proportionality, flexible parametric survival models^12^ were used to smooth survival curves and estimate survival differences during a 5-year period, which is the approximate follow-up if patients were enrolled midway through the recruitment period and remained in follow-up at the study end. Stata version 13.1 was used for all analyses.

This study is registered as an International Standard Randomised Controlled Trial, number ISRCTN 91273375.

### Role of the funding source

This study was led and funded by the UK Medical Research Council Clinical Trials Group. The trial was designed by members of the trial management group who reviewed and approved the protocol. The trial management group included representatives of the GCIG participating groups and the funding sources. Final decisions about trial conduct were the responsibility of chief investigators and funder. The trial management group were invited to comment on draft versions of this report, but responsibility for the report remained with the authors. ADC and AE had full access to all the raw data, and ADC had final responsibility for the decision to submit for publication.

## Results

**Figure 1:**
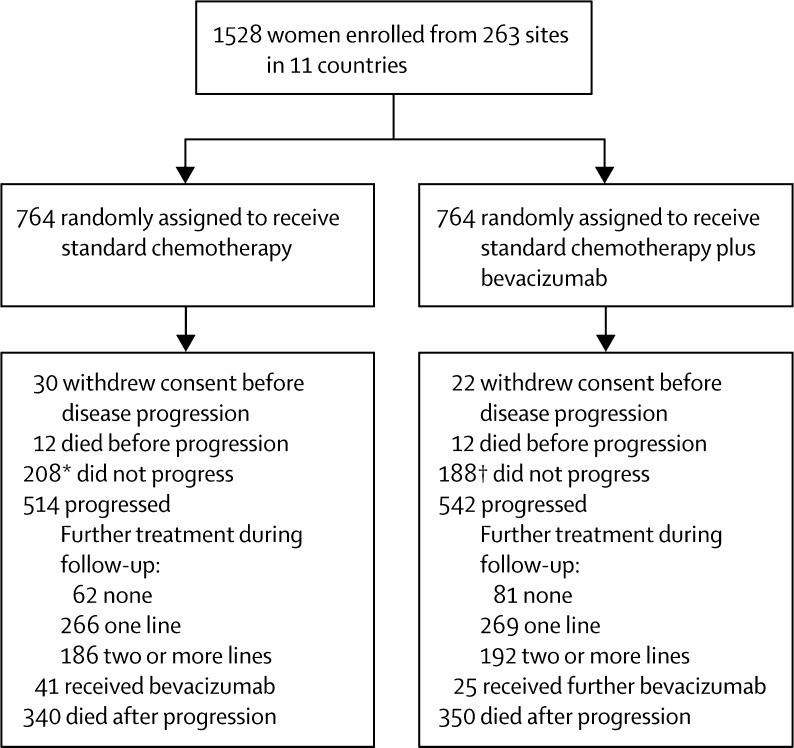
Trial profile *Includes 16 patients last seen more than 6 months before the end of the study. †Includes 11 patients last seen more than 6 months before the end of the study.

Between Dec 18, 2006, and Feb 16, 2009, seven GCIG groups recruited 1528 women with ovarian cancer from 263 centres across Europe, Canada, Australia, and New Zealand; these women were enrolled and randomly assigned to receive standard carboplatin and paclitaxel chemotherapy (n=764) or standard chemotherapy plus bevacizumab (n=764; figure 1). Median follow-up was 48·9 months (IQR 26·6–56·2), and follow-up ended on March 31, 2013. Patient baseline characteristics are summarised in appendix p 8; the median age of the patients was 57 years (IQR 50–64); 1415 (94%) of 1501 (excluding those with unknown performance status) had an ECOG performance status of 0 or 1; 1340 (89%) of 1502 (excluding those with cancer originating from multiple sites) had cancer of ovarian origin; 1054 (69%) had disease of serous histology; 175 (11%) had FIGO stage III, IIIA, or IIIB disease, and 1071 (70%) stage IIIC or IV disease. Following primary surgery, 395 (26%) patients had residual disease larger than 1 cm, 369 (24%) had visible disease up to 1 cm in diameter, and 734 (48%) had no visible residual disease. All randomised patients were included in analyses, and patient characteristics were well balanced between the groups (appendix p 8). Median follow-up was 48·6 months (IQR 24·3–56·0) in the standard chemotherapy group and 48·8 months (28·2–56·4) in the bevacizumab group, with shorter follow-up durations of 29·0 months (14·1–50·7) and 38·9 months (21·1–52·5), respectively, for high-risk patients.

The patient subgroup at high risk of progression consisted of 502 (33%) of 1528 patients. Their median age was 60 years (IQR 52–66) and 60 (12%) had cancer of primary peritoneal origin (compared with 106 [7%] of all enrolled patients overall). Most of the high-risk patients (381 [76%]) had serous-type ovarian cancer, and the group included 30 (6%) patients who did not undergo debulking surgery.

**Figure 2:**
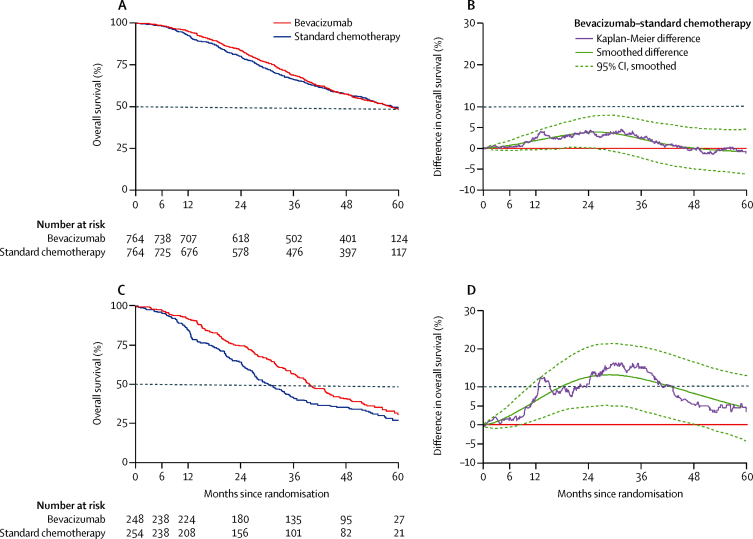
Overall survival (A) Overall survival in all patients. (B) Difference in overall survival between all patients in the two groups. (C) Overall survival in high-risk patients. (D) Difference in overall survival between high-risk patients in the two groups.

In total, 714 patients (47%) died during the study: 352 (46%) of those in the chemotherapy group and 362 (47%) of those in the chemotherapy plus bevacizumab group (table 1). The difference in overall survival between randomised groups was neither clinically nor statistically significant (log-rank test p=0·85), although non-proportionality was evident (p=0·02). Figure 2A shows the Kaplan-Meier survival curves for the two groups. Over time, the largest absolute difference in survival was less than 5%, occurring around 2 years after enrolment and favouring patients who received bevacizumab (figure 2B). Because the evidence of non-proportionality renders a hazard ratio difficult to interpret, we estimated restricted mean survival in each group. Restricted mean survival was 44·6 months (95% CI 43·2–45·9) for women in the chemotherapy group and 45·5 months (44·2–46·7) in the chemotherapy plus bevacizumab group (table 1).

Of the 502 high-risk patients, 332 (66%) died, including 174 (69%) of 254 in the chemotherapy group and 158 (54%) of 248 in the bevacizumab group (table 1). Evidence suggested longer overall survival in those who had received bevacizumab (p=0·03, figure 2C) but evidence of non-proportional hazards (p=0·01) meant that the hazard ratio was difficult to interpret. Restricted mean overall survival time was 39·3 months (95% CI 37·0–41·7) for the bevacizumab group and 34·5 months (32·0–37·0) for the chemotherapy group (log-rank p=0·03; table 1). The absolute difference in survival exceeded 10% after 2 years, and remained at 4·4% (95% CI −4·1 to 12·9) at 5 years (figure 2D). However, in non-high-risk patients, the restricted mean survival time did not differ significantly between the two treatment groups (49·7 months [95% CI 48·3–51·1]) in the standard chemotherapy group vs 48·4 months [47·0–49·9] in the bevacizumab group; p=0·20).

**Figure 3:**
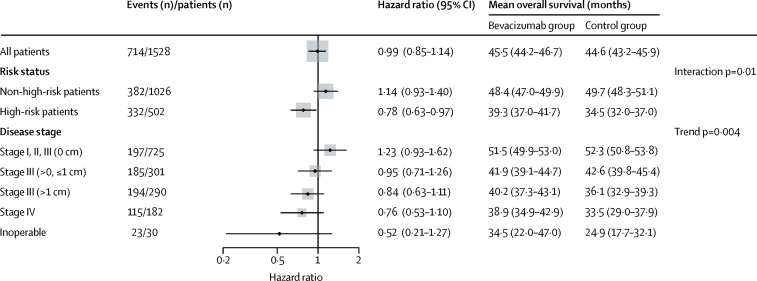
Treatment effect on overall survival by disease status at enrolment

Further analyses of survival by stage, residual disease burden, and risk of recurrence showed a benefit from bevacizumab with worsening prognostic factors (figure 3). Similar patterns were also noted for progression-free survival (p=0·014 for stage, p=0·005 for high risk; appendix p 12).

No benefit of bevacizumab was reported for other predefined poor-prognosis tumour types (table 2). Some baseline imbalance was recorded within subgroups, which is most likely a consequence of small numbers and is unlikely to have affected the results (appendix p 9). The mortality rate in all three subgroups was lower than in the overall trial population, especially in patients with low-stage high-grade disease (table 2). No evidence of difference between treatment groups was recorded within these subgroups (table 2).

In our extension of the previously reported quality-of-life analysis, now including data up to the predefined timepoint of week 76, in patients without disease progression, global quality of life did not differ between those who received standard chemotherapy and those receiving bevacizumab at week 76 (p=0·43, appendix p 9). In further exploratory analyses, a clinically small difference was recorded in patients receiving bevacizumab who were in the non-high-risk group relative to patients not receiving bevacizumab (−5·1 points, 95% CI −9·4 to −0·7; p=0·02), whereas a small and insignificant benefit (+4·3 points, 95% CI −4·9 to 13·4; p=0·36) relative to patients not receiving bevacizumab was noted in high-risk patients. A sensitivity analysis of missing data suggested that these findings were robust (appendix p 9).

The primary analysis of progression-free survival was previously reported when 759 patients had experienced disease progression or died (392 in the standard chemotherapy group and 367 in the bevacizumab group).^6^ Since these primary analyses, a further 321 patients have subsequently progressed or died without progression (134 in the standard chemotherapy group and 187 in the bevacizumab group) for a total of 1080 progression events or deaths. The overall difference in progression-free survival between randomised groups was no longer statistically significant (table 1; appendix p 11). However, in high-risk patients, a significant benefit remains (p=0·001) with strong evidence of non-proportional hazards (p<0·0001), and longer mean restricted progression-free survival in the bevacizumab group than in the chemotherapy group (table 1).

Most adverse events that occurred in the trial have been previously reported: bevacizumab was associated with an increase in grade 1–2 mucocutaneous bleeding (271 [36%] patients in the bevacizumab group *vs* 55 [7%] patients in the standard chemotherapy group), grade 2 or worse hypertension (136 [18%] *vs* 16 [2%]), grade 3 or worse thromboembolic events (51 [7%] *vs* 23 [3%]), and grade 3 or worse gastrointestinal perforations (ten [1%] *vs* three [<1%]).^6^ During extended follow-up of overall survival, one further treatment-related grade 3 event (gastrointestinal fistula in a bevacizumab-treated patient), three grade 2 treatment-related events (cardiac failure, sarcoidosis, and foot fracture, all in bevacizumab-treated patients), and one grade 1 treatment-related event (vaginal haemorrhage, in a patient treated with standard chemotherapy) were also reported.

## Discussion

The results of ICON7 show that bevacizumab did not improve overall survival in the intention-to-treat population of women randomly assigned to receive it in conjunction with chemotherapy, although heterogeneity of benefit was observed dependent on residual disease burden before treatment. Although the overall difference between treatment groups was not statistically significant, non-proportionality was recorded, despite the fact that the magnitude of the change over time was not clinically meaningful. However, in a preplanned analysis, women at high risk of disease progression had a significant improvement in overall survival with the addition of bevacizumab to standard chemotherapy. A similar improvement in overall survival was also reported in high-risk patients (>1 cm residual tumour) in GOG-218 (HR 0·73 in ICON7, HR 0·86 in GOG-218), with some differences as expected because of varying post-progression treatment strategies, in particular greater use of bevacizumab (ie, more participants receiving the drug) in GOG-218 patients. These findings are relevant since, in practice, bevacizumab use has become focused on the high-risk patient group.

In addition to the contrast between high-risk and non-high-risk patients, there was a clear association between increasing disease severity and a stronger beneficial effect of bevacizumab (figure 3). To our knowledge, this is the first study with bevacizumab to show this trend in a single trial. These observations provide a clinical framework for the appropriate use of bevacizumab in ovarian cancer that is consistent with the biological requirement for angiogenesis in growing tumours, and a hypothetical framework to explain this effect biologically. Our data suggest that a residual physical tumour burden, presumably producing VEGF, is necessary to enable bevacizumab to exert its effect on the tumour microenvironment. Other trials in ovarian cancer have also reported an overt benefit in women with a measurable (higher) disease burden following recurrence, in both platinum-sensitive^7^ and platinum-resistant settings.^8^

These final results complement the findings of the earlier primary progression-free survival analysis,^6^ with a benefit of bevacizumab recorded in women with advanced-stage suboptimally debulked disease. The magnitude and duration of benefit in this group is both clinically and statistically significant. The primary progression-free survival analyses also showed similar outcomes in GOG-218 patients and the high-risk patient group of ICON7.^5^, ^6^ The updated progression-free survival analysis reported here showed a reduced overall effect, with greater elapsed time from treatment with bevacizumab, but the earlier results remain the primary pre-specified analysis.

Our data also identify patients who might not benefit from bevacizumab in the first-line setting. Women with early-stage (FIGO stage I/II) disease, even if judged to be high risk on the basis of grade or clear cell histology, do not seem to benefit. Women with optimally debulked (<1 cm) stage III disease also had no benefit. Furthermore, a small reduction in overall quality of life was recorded in non-high-risk patients treated with bevacizumab. Our trial included patients with all stages of newly diagnosed epithelial ovarian, fallopian tube, and primary peritoneal cancer for whom postoperative chemotherapy would usually be indicated. It also included 30 patients in whom primary, and subsequent, debulking surgery was regarded as unlikely to be in the patient's best interests.

Three subgroups based on tumour type were also predefined: clear cell, low-grade serous, and high-risk low-stage cancer. Women with clear cell carcinoma comprised 10% of the study population. This histology was previously thought to confer a substantially worse outcome than other tumour subtypes but these patients did surprisingly well in our trial, with a 72% survival after a median of 51 months' follow-up, with no benefit from bevacizumab. Patients with low-grade serous cancer also did not benefit from the addition of bevacizumab, although a major limitation of this assessment is the absence of central pathology review for these tumours. Patients with low-stage high-risk tumours also did not benefit from the addition of bevacizumab. For all three subgroup analyses, the numbers of patients were small and statistical power to detect differences was low.

To reflect on the choice of outcome measures for the ICON7 trial is pertinent. The primary outcome measure was progression-free survival, but the trial was also a-priori structured to assess overall survival. To assess this outcome measure needed a further 3 years of follow-up after the primary progression-free survival analysis, but also simplified aspects of trial design, such as placebo control and independent masked radiology review, which are essential when progression-free survival is the only outcome measure. The primary analysis in 2011 showed a significant progression-free survival benefit in the intention-to-treat population, which was most pronounced in women at high risk of progression.^6^ Over time, the effect closely followed bevacizumab treatment, with the maximum benefit coinciding precisely with duration of treatment (appendix p 11). Notably, the overall survival difference outlasts the duration of bevacizumab exposure and points to a durable benefit in the high-risk group (figure 2D), raising the possibility of additional benefit to high-risk patients from further extension of treatment duration, which is the subject of ongoing research in the BOOST trial (NCT01462890). Following disease progression in ICON7, the pattern of further treatment was similar in both randomised groups, with little use of further bevacizumab in either group.

Questions about the optimum timing of bevacizumab therapy in the trajectory of a woman's disease remain. Should it be considered at initial presentation, time of platinum-sensitive recurrence, or after the development of platinum resistance? Bevacizumab has shown a significant progression-free survival benefit in all these settings,^5^, ^6^, ^7^, ^8^ and an overall survival benefit in some settings too.^6^, ^13^, ^14^ Our data strongly support early use of bevacizumab, based on risk and disease burden. Whether or not bevacizumab can be used beyond progression in this indication, and whether or not treatment can be repeated, remains an intriguing question that is being addressed in the MITO16MANGO2b trial (NCT01802749); with studies in colorectal cancer^15^ and breast cancer^16^ having already provided evidence of benefit.

From a societal perspective, there are strong and sometimes opposing views about the costs and cost–benefit ratio of bevacizumab. The JGOG-316 trial^17^ reported a similar overall survival benefit without bevacizumab from the use of weekly paclitaxel, whereas GOG-262^18^ suggests that the effect of bevacizumab may be attenuated by such a strategy. Full economic analyses related to our trial are ongoing and will be reported separately. Interestingly, the small reduction in quality of life associated with bevacizumab that was reported after 54 weeks^19^ was smaller still by week 76 and was not statistically significant. The ability to predict which patients will benefit most is clearly important and could have the power to change the cost-effectiveness of treatment substantially by not treating patients with little chance of benefit. Our data suggest a simple and pragmatic clinical algorithm based on residual disease. Many studies are underway to identify a biomarker signature of response or resistance in patients in our trial. Collinson and colleagues^20^ and Backen and colleagues^21^ have presented biomarker strategies with potentially predictive approaches, whereas Gourley and colleagues^22^ have reported that bevacizumab might disadvantage women with an immunologically active subtype and Winterhoff and coworkers have reported benefit for women with mesenchymal-subtype disease.^23^ These findings all need to be validated in independent datasets.

The addition of bevacizumab to chemotherapy is an important step forward in integrating biological agents with conventional chemotherapy in ovarian cancer. This trial provides evidence of a benefit in poor-prognosis patients. Future studies will refine important questions of biological prediction, duration, and rechallenge.

## Figures and Tables

**Table 1:** Primary analysis of overall survival and updated analysis of progression-free survival

	**All patients**		**High-risk patients**	
	Standard therapy (n=764)	Bevacizumab (n=764)	Standard therapy (n=254)	Bevacizumab (n=248)
**Overall survival**				
Follow-up duration (months)	48·6 (24·3–56·0)	48·8 (28·2–56·4)	29·0 (14·1–50·7)	38·9 (21·1–52·5)
Deaths	352 (46%)	362 (47%)	174 (69%)	158 (64%)
Median overall survival (months; 95% CI)	58·6 (53·5–67·5)	58·0 (52·4–66·9)	30·2 (27·0–34·3)	39·7 (36·0–44·2)
Log-rank test p value	p=0·85		p=0·03	
HR (95% CI)	0·99 (0·85–1·14)		0·78 (0·63–0·97)	
Non-proportionality p value*	p=0·02		p=0·01	
(Restricted) mean survival time (months; 95% CI)†	44·6 (43·2–45·9)	45·5 (44·2–46·7)	34·5 (32·0–37·0)	39·3 (37·0–41·7)
Restricted mean survival time difference (95% CI)	0·9 (−0·8 to 2·6)		4·8 (1·5–8·1)	
**Progression-free survival**				
Follow-up duration (months)	16·3 (8·8–48·4)	19·4 (12·7–45·3)	10·1 (7·7–18·2)	15·6 (9·9–21·7)
Disease progression	526 (74%)	554 (73%)	228 (90%)	223 (90%)
Median progression-free survival (months; 95% CI)	17·5 (15·7–18·7)	19·9 (19·1–22·0)	10·5 (9·3–12·0)	16·0 (14·2–17·8)
Log-rank test p value	p=0·25		p=0·001	
HR (95% CI)	0·93 (0·83–1·05)		0·73 (0·61–0·88)	
Non-proportionality p value*	p<0·0001		p<0·0001	
(Restricted) mean survival time (months; 95% CI)†	27·7 (26·1–29·2)	29·2 (27·7–30·7)	15·9 (14·1–17·7)	20·0 (18·1–21·8)
Restricted mean survival time difference (95% CI)	1·6 (−0·6 to 3·7)		4·1 (1·4–6·7)	

Data are median (IQR) or n (%), unless otherwise indicated. HRs, p values, and survival time differences are for differences between the standard therapy and bevacizumab groups. HR=hazard ratio.

* Grambsch-Therneau test.

† Restricted at 5 years.

**Table 2:** Overall survival in predefined subgroups

	Clear cell tumours*		**Low-stage high-grade tumours**		**Low-grade serous tumours**	
	Standard therapy (n=77)	Bevacizumab (n=82)	Standard therapy (n=75)	Bevacizumab (n=67)	Standard therapy (n=49)	Bevacizumab (n=31)
Follow-up duration (months)	52·5 (29·0–57·5)	50·7 (28·2–57·9)	55·3 (49·1–60·6)	55·4 (51·2–61·6)	50·5 (28·2–55·1)	55·3 (47·9–62·0)
Deaths	20 (26%)	24 (29%)	6 (8%)	9 (13%)	13 (27%)	7 (23%)
Log-rank test p value	p=0·74		p=0·44		p=0·60	
HR (95% CI)	1·09 (0·64–1·88)		1·49 (0·53–4·20)		0·78 (0·31–1·97)	
Non-proportionality p value†	p=0·58		p=0·002		p=0·07	
(Restricted) mean survival time (months; 95% CI)‡	48·0 (43·9–52·2)	47·6 (43·6–51·6)	56·2 (51·5–60·9)	57·5 (55·7–59·4)	50·4 (45·6–55·2)	50·5 (43·9–57·0)
Restricted mean survival time difference (95% CI)	−0·4 (−6·1 to 5·3)		1·3 (−3·7 to 6·4)		0·1 (−7·9 to 8·0)	

Data are median (IQR) or n (%), unless otherwise indicated. HRs, p values, and survival time differences are for differences between the standard therapy and bevacizumab groups.

* The clear cell tumour group includes some patients with mixed histology.

† Grambsch-Therneau test.

‡ Restricted at 5 years.
