# Supplementary material for: Standard chemotherapy with or without bevacizumab for women with newly diagnosed ovarian cancer (ICON7): overall survival results of a phase 3 randomised trial
Source: Lancet Oncol. 2015 Aug;16(8):928–36. doi: 10.1016/S1470-2045(15)00086-8 (PMC4648090; doi:10.1016/S1470-2045(15)00086-8)

THE LANCET Oncology

Supplementary appendix

This appendix formed part of the original submission and has been peer reviewed. We post it as supplied by the authors.

Supplement to: Oza AM, Cook AD, Pfisterer J, et al, for the ICON7 trial investigators. Standard chemotherapy with or without bevacizumab for women with newly diagnosed ovarian cancer (ICON7): overall survival results of a phase 3 randomised trial. *Lancet Oncol* 2015; published online June 24. http://dx.doi.org/10.1016/S1470-2045(15)00086-8.

Supplementary material

List of participating sites

Institute (Country) Investigator [Number of patients]

The Norwegian Radium Hospital (NOR) Dr. Gunnar Kristensen [37]
University College London Hospital (GBR) Dr. Jonathan Ledermann [28]
Universitaetsklinikum Ulm (DEU) Pr. Rolf Kreienberg [26]
Hôpital Tenon (FRA) Dr. Frédéric Selle [25]
HSK Dr. Horst Schmidt Klinik (DEU) Pr. Andreas du Bois [23]
St James's University Hospital (GBR) Dr. Timothy Perren [22]
Univ. Health Network-OCI (CAN) Dr. Amit Oza [20]
Helsinki University Hospital (FIN) Dr. Arto Leminen [20]
Edinburgh Cancer Research Centre (GBR) Dr. Charlie Gourley [20]
Royal Surrey County Hospital (GBR) Dr. Sharadah Essapen [20]
Odense Universitetshospital (DEN) Jørn Herrstedt [18]
Centre Alexis Vautrin-Brabois (FRA) Dr. Béatrice Weber [18]
Christie Hospital (GBR) Dr. Jurgees Hasan [17]
Clatterbridge Centre for Oncology (GBR) Dr. John Green [17]
Mount Vernon Hospital (GBR) Pr. Gordon Rustin [17]
Campus Virchow Klinikum (DEU) Pr. Jalid Sehouli [16]
Universitaetsklinikum Essen (DEU) Dr. Pauline Wimberger [16]
CHUQ - Hotel Dieu de Québec (CAN) Dr. Marie Plante [15]
Centre Catherine de Sienne (FRA) Dr. Alain Lortholary [15]
London Regional Cancer Program (CAN) Dr. Monique Bertrand [14]
St. Vincentius Kliniken gAG (DEU) Dr. Anne Staehle [14]
Churchill Hospital (GBR) Pr. Bass Hassan [14]
John Vane Science Center (GBR) Pr. Iain McNeish [14]
Royal Women's Hospital (AUS) Pr. Michael Quinn [13]
Centre François Baclesse (FRA) Dr. Florence Joly [13]
Hammersmith Hospital (GBR) Dr. Sarah Blagden [13]
St Oslavs Hospital (NOR) Dr. Ingrid Baasland [13]
Universitaetsklinikum Schleswig-Holstein Kiel (DEU) Dr. Felix Hilpert [12]
Institut Gustave Roussy (FRA) Dr. Catherine Lhomme [12]
Royal Cornwall Hospital (GBR) Dr. Nigel Bailey [12]
Queen Elizabeth The Queen Mother Hospital (GBR) Dr. Justin Waters [12]
Haukeland Universitetssykehus (NOR) Dr. Harald Helland [12]
Lund University Hospital (SWE) Kjell Bergfeldt [12]
Klinikum Bremen-Mitte (DEU) Pr. Willibald Schroeder [11]
Med. Hochschule Hannover (DEU) Pr. Henning Kuehnle [11]
Universitaetsklinikum Tübingen (DEU) Pr. Erich Solomayer [11]
Universitaetsklinikum Giessen u. Marburg GmbH, S (DEU) Dr. Klaus Baumann [11]
Universitätskrankenhaus Hamburg-Eppendorf (DEU) Dr. Sven Mahner [11]
St Mary's Hospital (GBR) Dr. Yoodhivir Nagar [11]
Herlev Hospital (DEN) Hanne Havsteen [10]
Klinikum Kassel (DEU) Dr. Hans Urbanczyk [10]
Guy's Hospital (GBR) Dr. Peter Harper [10]
Newcastle General Hospital (GBR) Dr. Graham Dark [10]
CHUM - Hôpital Notre-Dame (CAN) Dr. Diane Provencher [9]
Aalborg Sygehus (DEN) Dr. Bente Lund [9]
Diakonissenanstalt (DEU) Dr. Horst Ostertag, [9]
Ev. Waldkrankenhaus Spandau (DEU) Dr. Jochen Potenberg [9]
Klinikum Grosshadern (DEU) Dr. Alexander Burges [9]
Klinikum der Universitaet zu Koeln (DEU) Dr. Joachim Schneider [9]
Universitaetsklinikum Schleswig-Holstein Lubeck (DEU) Pr. Marc Thill [9]
Institut Bergonié (FRA) Dr. Anne Floquet [9]
Cheltenham General Hospital (GBR) Dr. Radi Counsell [9]
Kent Oncology Centre (GBR) Dr. Jeff Summers [9]
Royal Marsden Hospital Sutton (GBR) Pr. Stan Kaye [9]
Weston Park Hospital (GBR) Dr. Simon Pledge [9]
Radiumhemmet (SWE) Dr. Bengt Tholander [9]

Prince of Wales Hospital (AUS) Pr. Michael Friedlander [8]
Herning Sygehus (DEN) Dr. Nina Keldsen [8]
Rigshospitalet (DEN) Dr. Svend Aage Engelholm [8]
Albertinen Krankenhaus (DEU) Pr. Martin Carstensen [8]
Klinikum St. Georg, Franziskus Hospital Harderbe (DEU) Dr. Michael Hoedemaker [8]
Oulu University Hospital (FIN) Dr. Ulla Puistola [8]
Centre Jean Perrin (FRA) Dr. Xavier Durando [8]
Centre Léon Bérard (FRA) Dr. Isabelle Ray-Coquard [8]
University Hospital (SWE) Dr. Per Rosenberg [8]
Royal Hobart Hospital (AUS) Dr. Rob McIntosh [7]
Sir Charles Gairdner Hospital (AUS) Dr. Martin Buck [7]
BCCA - Fraser Valley (CAN) Dr. Ursula Lee [7]
Tom Baker Cancer Centre (CAN) Dr. Prafull Ghatage [7]
Klinikum d. Ernst-Moritz-Arndt-Universitaet (DEU) Dr. Antje-K. Belau [7]
Klinikum d. Johannes Gutenberg-Universitaet (DEU) Dr. Marcus Schmidt [7]
Klinikum der J.W. Goethe-Universitaet (DEU) Dr. Lars Hanker [7]
St. Johannes Hospital (DEU) Dr. Georg Kunz [7]
Universitaetsklinikum Carl Gustav Carus (DEU) Dr. Ulrich Canzler [7]
Universitätsklinikum Jena (DEU) Pr. Ingo Runnebaum [7]
Turku University Hospital (FIN) Dr. Seija Grenman [7]
CRLC Val d'Aurelle (FRA) Dr. Michel Fabbro [7]
Centre d'Oncologie de Gentilly (FRA) Dr. Dominique Spaeth [7]
Institut Curie (FRA) Dr. Paul-Henri Cottu [7]
Institut Sainte Catherine (FRA) Dr. Gaëtan De Rauglaudre [7]
Derbyshire Royal Infirmary (GBR) Dr. Mojca Persic [7]
Queen Elizabeth Hospital (GBR) Dr. Raj Naik [7]
The Deanesly Centre (GBR) Dr. Rozenn Allerton [7]
Stavanger University Hospital (NOR) Dr. Bent Fiane [7]
Christchurch Hospital (NZ) Dr. Bernie Fitzharris [7]
Border Medical Oncology (AUS) Dr. Christophee Steer [6]
Centre Hospitalier Universitaire de Sherbrooke (CAN) Dr. Paul Bessette [6]
Juravinski Cancer Centre (CAN) Dr. Laurie Elit [6]
McGill University (CAN) Dr. Walter Gotlieb [6]
Gemeinschaftspraxis f. Frauenheilkunde u. Geburt (DEU) Dr. Wolfgang Dietz [6]
Henriettenstiftung (DEU) Dr. Iris Schrader [6]
Karolinen-Hospital Hüsten (DEU) Dr. Norbert Peters [6]
Klinikum Schaumburg Kreiskrankenhaus Stadthagen (DEU) Dr. Johannes Feltz-Suessenbach [6]
Klinikum Suedstadt d. Hansestadt Rostock (DEU) Pr. Bernd Gerber [6]
Kreikrankenhaus Torgau "Johann Kentmann" gGmbH (DEU) Dr. Eike Simon [6]
St. Josefsklinik (DEU) Dr. Dietmar Schwörer [6]
Staedt. Klinikum Karlsruhe (DEU) Pr. Hans-Ulrich Ulmer [6]
Staedt. Klinikum Lueneburg (DEU) Dr. Eric Boetel [6]
Instituto Valenciano de Oncología (ESP) Dr. Andrés Poveda [6]
Hôpital Saint-Louis (FRA) Pr. Jean-Louis Misset [6]
Ipswich Hospital NHS Trust (GBR) Dr. Jamey Morgan [6]
Ninewells Hospital (GBR) Dr. Michelle Ferguson [6]
Velindre Hospital (GBR) Dr. Malcolm Adams [6]
Credit Valley Hospital (CAN) Dr. Leonard Kaizer [5]
QEII HSC Victoria Site (CAN) Dr. Robert Grimshaw [5]
Elisabeth-Krankenhaus (DEU) Pr. Stefan Niesert, [5]
Klinikum Innenstadt, LMU Muenchen (DEU) Pr. Harald Sommer [5]
Kreiskrankenhaus Freudenstadt (DEU) Dr. Andreas Kuznik [5]
Kreiskrankenhaus Waiblingen (DEU) Edina Sulea [5]
Otto-von-Guericke-Universitaet (DEU) Dr. Joachim Bischoff [5]
Staedt. Klinikum Guetersloh (DEU) Dr. Joachim Hulde [5]
Hospital Clinic Barcelona (ESP) Dr. Pere Gascón [5]
CHD Les Oudairies (FRA) Dr. Franck Priou [5]
Clinique Armoricaine de Radiologie (FRA) Dr. Anne-Claire Hardy-Bessard [5]
Addenbrookes Hospital (GBR) Dr. Helena Earl [5]
Poole Hospital (GBR) Dr. Richard Osbourne [5]
Wexham Park Hospital (GBR) Dr. Marcia Hall [5]
Allan Blair Cancer Centre (CAN) Dr. Haji Chalchal [4]
Cross Cancer Institute (CAN) Dr. Valerie Capstick [4]
Dietrich-Bonhoeffer-Klinikum (DEU) Katrin Borkowski [4]

Ev. Krankenhaus Koeln-Kalk GmbH (DEU) Dr. Wolfgang Maurer [4]
Hämatologisch-Onkologische Schwerpunktpraxis (DEU) Dr. Helmut Forstbauer [4]
Katholisches Klinikum, St. Vincenz u. Elisabeth (DEU) Pr. Wolfgang Wiest [4]
Klinikum Göttingen, Georg-August-Universität (DEU) Pr. Günter Emons [4]
Klinikum Itzehoe (DEU) Dr. Britta Seifert [4]
Klinikum Mannheim gGmbH, Universitaetsklinikum (DEU) Pr. Marc Suetterlin [4]
Malteser Krankenhaus (DEU) Dr. Martina Gropp [4]
Gemeinschaftpraxis, Braunschweig (DEU) Dr. Ralf Lorenz [4]
Städtisches Klinikum Dessau (DEU) Dr. Inken Achtert [4]
Städtisches Klinikum Magdeburg, Standort Olvenst (DEU) Dr. Erika Kettner [4]
Universität Erlangen-Nürnberg (DEU) Pr. Matthias W. Beckmann [4]
Hospital Clínica Universitario de Valencia (ESP) Dr. Andrés Cervantes [4]
Hospital Valle Hebrón (ESP) Dr. José María del Campo [4]
ICO - Hospital Josep Trueta (ESP) Dr. Miguel Beltrán [4]
ICO Durán y Reynals (ESP) Dr. Ana Oaknin [4]
Centre Antoine Lacassagne (FRA) Dr. Véronique Mari [4]
Centre Oscar Lambret (FRA) Dr. Anne Lesoin [4]
Centre Paul Papin (FRA) Dr. Rémy Delva [4]
Centre René Gauducheau (FRA) Dr. Dominique Berton-Rigaud [4]
Hôpital Hôtel Dieu (FRA) Pr. Eric Pujade-Lauraine [4]
Hôpitaux Civils de Colmar - Centre Hospitalier L (FRA) Dr. Jean-Claude Barats [4]
Gartnavel General Hospital (GBR) Dr. Nick Reed [4]
James Cook University Hospital (GBR) Dr. Sath Gokul [4]
Leicester Royal Infirmary (GBR) Dr. Paul Symonds [4]
Queens Hospital (GBR) Dr. Mojca Persic [4]
Royal Marsden Hospital London (GBR) Pr. Martin Gore [4]
Canberra Hospital (AUS) Dr. Alison Davis [3]
Royal North Shore Hospital (AUS) Dr. Sally Baron-Hay [3]
Sydney Cancer Centre (AUS) Dr. Philip Beale [3]
BCCA-Kelowna (CAN) Dr. Susan Ellard [3]
Ev. Jung-Stilling-Krankenhaus (DEU) Dr. Frank Lauber [3]
Gemeinschaftspraxis , Hildesheim (DEU) Dr. Christoph Uleer [3]
Helios Kliniken GmbH, Klinikum Buch (DEU) Dr. Elke Keil [3]
Herz-Jesu-Krankenhaus Fulda gGmbH (DEU) Dr. Dietrich Mosch [3]
Johanniter Krankenhaus Bonn (DEU) Pr. Uwe-Jochen Göhring [3]
Klinikum Darmstadt (DEU) PD Dr. Sven Ackermann [3]
Klinikum Rosenheim (DEU) Dr. Thomas Beck [3]
Klinikum Ernst-von-Bergmann Potsdam (DEU) Dr. Friedrich Dreßler [3]
Klinikum des Landkreises Deggendorf (DEU) Dr. Ronaldo Stuth [3]
Kreiskrankenhaus Sigmaringen (DEU) Dr. Peter Krezdorn [3]
Onkolog. Gemeinschaftspraxis (DEU) Pr. Hans Tesch [3]
Praxis Dr. Beha, Amberg (DEU) Dr. Michaela Beha [3]
Praxis Haematologie u. internistische Onkolog, Wuppertal (DEU) Dr. Werner Fett [3]
Staedt. Kliniken Esslingen (DEU) Dr. Cornelia Kurz [3]
Staedt. Kliniken Neuss, Lukaskrankenhaus (DEU) Pr. Hans-Georg Schnuerch [3]
Staedt. Klinikum Neunkirchen (DEU) Dr. Georg-Peter Breitbach [3]
Universitätsklinikum Bonn (DEU) Dr. Martin Pölcher [3]
Hospital Miguel Servet (ESP) Dr. Ana Herrero [3]
Hospital Ramón y Cajal (ESP) Dr. Antonio González [3]
Hospital Sant Pau (ESP) Dr. Belén Ojeda [3]
Centre Claudius Regaud (FRA) Dr. Laurence Gladieff [3]
Hôpital Civil (FRA) Pr. Jean-Emmanu Kurtz [3]
Aberdeen Royal Infirmary (GBR) Radha Todd [3]
Broomfield Hospital (GBR) Dr. Saad Tahir [3]
Nottingham City Hospital (GBR) Dr. Steven Chan [3]
Princess Royal Hospital (GBR) Dr. Penelope O'Neill [3]
Wellington Hospital (NZ) Dr. Anne O'Donnell [3]
University Hospital (SWE) Pr. Bengt Sorbe [3]
Medical Oncology Box Hill Hospital (AUS) Dr. Geraldine Goss [2]
Mercy Hospital for Women (AUS) Pr. Danny Rischin [2]
Newcastle Mater Misericordiae Hospital (AUS) Dr. Tony Bonaventura [2]
Royal Brisbane Hospital (AUS) Dr. Nicole McCarthy [2]
Wesley Medical Centre (AUS) Dr. Paul Vasey [2]
Women's Health Centre Royal Adelaide Hospital (AUS) Dr. Margaret Davy [2]

BCCA - Vancouver Cancer Centre (CAN) Paul Hoskins [2]
The Moncton Hospital (CAN) Dr. Asif Shaikh [2]
Asklepios Klinik Nord, Campus Heidberg (DEU) Dr. Peter Müller [2]
Diakonissenkrankenhaus Karlsruhe (DEU) Dr. Gerhard Deutsch [2]
Elblandkliniken Meissen-Radebeul (DEU) Dr. Barbara Richter [2]
Ev. Diakoniewerk Friederikenstift Hannover (DEU) Dr. Joerg Gade [2]
Klinikum Dorothea-Christiane-Erxleben Quedlinburg (DEU) Dr. Otto Boldt [2]
Klinikum Bayreuth (DEU) Pr. Augustinus Tulusan [2]
Klinikum Chemnitz (DEU) Dr. Petra Krabisch [2]
Klinikum Coburg (DEU) Dr. Hermann Zoche [2]
Klinikum Fürth (DEU) Pr. Volker Hanf [2]
Klinikum Lahr (DEU) Dr. Daniel Pfisterer [2]
Krankenhaus am Rosarium (DEU) Dipl.-Med Annette Müller [2]
Kreiskrankenhaus Leonberg (DEU) Dr. Harald Wolf [2]
Leopoldina-Krankenhaus Schweinfurt (DEU) Pr. Michael Weigel [2]
Marien-Hospital Witten (DEU) Dr. Felix Bartzsch [2]
Marienhaus Klinikum, St. Elisabeth Krankenhaus (DEU) Pr. Richard Berger [2]
Marienhospital Stuttgart (DEU) Dr. Heinrich Stehle [2]
Oberschwabenklinik, Krankenhaus St. Elisabeth (DEU) Dr. Gerhard Fischer [2]
Ostalb-Klinikum Aalen (DEU) Dr. Carina Paschold [2]
Paracelsus-Klinik Henstedt-Ulzburg (DEU) Dr. Barbara Jahns [2]
Praxis Vehling-Kaiser Landshut (DEU) Dr. Ursula Vehling-Kaiser [2]
Praxisklinik Krebsheilkunde Berlin (DEU) Dr. Peter Klare [2]
Robert-Bosch-Krankenhaus (DEU) Pr. Walter Aulitzky [2]
St. Elisabethen-Krankenhaus (DEU) Dr. Brigitte Schuhman [2]
St. Josefs-Hospital (DEU) Pr. Gerald Hoffmann [2]
St. Joseph-Stift (DEU) Pr. Christiane Frantzen [2]
St. Marien-Krankenhaus (DEU) Dr. Matthias Stamm [2]
St. Vincenz Krankenhaus (DEU) Dr. Peter Scheler [2]
Stadtkrankenhaus Worms (DEU) Pr. Thomas Hitschold [2]
Staedt. Krankenhaus Frankfurt-Hoechst (DEU) Pr. Volker Moebus [2]
Universitaetsklinikum Freiburg (DEU) Pr. Annette Hasenburg [2]
Hospital Universitario La Paz (ESP) Dr. Andrés Redondo [2]
Centre Henri Becquerel (FRA) Dr. Cécile Guillemet [2]
Hôpital André Mignot (FRA) Dr. Jean-Franço Geay [2]
Hôpital Cochin (FRA) Pr. François Goldwasser [2]
Huddersfield Royal Infirmary (GBR) Dr. Barbara Crosse [2]
Queens Hospital Romford (GBR) Dr. Mary Quigley [2]
Plymouth Oncology Centre (GBR) Dr. Dennis Yiannakis [2]
St Georges Hospital (GBR) Dr. Fiona Lofts [2]
University Hospital Coventry and Warwickshire NH (GBR) Dr. Clive Irwin [2]
Dunedin Hospital (NZ) Dr. David Perez [2]
Cabrini Institute (AUS) Dr. Garry Richardson [1]
Lismore Base Hospital (AUS) Dr. Adam Boyce [1]
Algoma District Program (CAN) Dr. Silvana Spadafora [1]
CancerCare Manitoba (CAN) Dr. Robert Lotocki [1]
Dr. H. Bliss Murphy Cancer Centre (CAN) Dr. Patti Power [1]
Asklepios Klinik Bad Oldesloe (DEU) Dr. Tobias Zeiser [1]
Augusta-Kranken-Anstalt Bochum (DEU) Pr. Dirk Behringer [1]
Klinikum Hanau (DEU) Dr. Thomas Müller [1]
Klinikum Offenbach (DEU) Dr. Dunja Reitz [1]
Klinikum am Steinenberg (DEU) Dr. Peter Krieger [1]
Klinikum d. Stadt Wolfsburg (DEU) Pr. Karl Ulrich Petry, [1]
Klinikverbund Südwest, Klinikum Sindelfingen-Böb (DEU) Dr. Erich Weiss [1]
Kreisklinik Ebersberg (DEU) Dr. Cornelia Hoess [1]
Kreiskrankenhaus Gifhorn (DEU) Dr. Viola Siegmund [1]
Kreiskrankenhaus Rottweil (DEU) Dr. Gerhard Bartzke, [1]
Medizinisches Zentrum Bonn Friedensplatz (DEU) Dr. Christian Kurbacher [1]
Missionsaerztliche Klinik Wuerzburg (DEU) Dr. Wibke Glueer [1]
Onkologische Gemeinschaftspraxis (DEU) Dipl.-Med. Steffen Dörfel [1]
Praxis Onkologie u. Hamatologie Freiburg (DEU) Dr. Norbert Marschner [1]
Universitätsfrauenklinik Giessen (DEU) Pr. Karsten Münstedt [1]
Hospital Marqués de Valdecilla (ESP) Dr. Ana de Juan [1]
Hospital Son Llatzer (ESP) Dr. Isabel Bover [1]

Hospital Universitario La Fe (ESP) Dr. Ana Santaballa [1]
Centre Hospitalier Régional Universitaire (CHRU) (FRA) Pr. Mohamed Hebbar [1]
Centre René Huguenin (FRA) Dr. Fawzia Mefti [1]
Clinique Jean Bernard SARL (FRA) Dr. Hugues Bourgeois [1]
Hôpital Européen Georges Pompidou (FRA) Dr. Eric Levy [1]
Hôpital Foch (FRA) Dr. Mehran Khatibi [1]
Hôpital Mont de Marsan (FRA) Dr. Jérôme Dauba [1]
Hôpital des Diaconesses (FRA) Dr. Anne Plantade [1]
Institut Jean Godinot (FRA) Pr. Hervé Cure [1]
Essex County Hospital (GBR) Dr. Alan Lamont [1]
Great Western Hospital (GBR) Dr. Amanda Horne [1]
North Devon District Hospital (GBR) Dr. Mark Napier [1]
Queen Elizabeth Hospital (GBR) Dr. Ahmed El-Modir [1]
Southampton General Hospital (GBR) Dr. Clare Green [1]
Yeovil District Hospital NHS Foundation (GBR) Dr. Geoffrey Sparrow [1]

List of contributors

AGO-OVAR group: Jacobus Pfisterer, Anja Krüger, Gabriele Elser
Ostalb-Klinikum, Frauenklinik, Aalen (Dr Carina Paschold); Praxis Dr. Beha, Amberg (Dr Michaela Beha) ; Karolinen-Hospital Hüsten, Frauenheilkunde u. Geburtshilfe, Arnsberg (Dr Norbert Peters) ; Klinikum Bayreuth GmbH, Frauenklinik (Pr Augustinus H. Tulusan) ; Campus Virchow Klinikum, Charite, Klinik f. Frauenheilkunde u. Geburtshilfe, Berlin (Pr Jalid Sehouli) ; Ev. Waldkrankenhaus Spandau, Innere Abteilung, Berlin (Dr Jochem Potenberg) ; HELIOS Kliniken GmbH, Klinikum Buch, Frauenklinik, Berlin (Dr Elke Keil) ; Praxisklinik, Berlin (Dr Peter Klare); Klinikverbund Südwest, Kliniken Böblingen, Frauenklinik (Dr Erich Weiss) ; Augusta-Kranken-Anstalt gGmbH, Klinik f. Hämatologie u. internistische Onkologie, Bochum (Pr Dirk Behringer) ; Malteser Krankenhaus, Gynäkologie u. Geburtshilfe, Bonn (Dr Martina Gropp) ; Universitätsklinikum, Zentrum f. Geburtshilfe u. Frauenheilkunde, Bonn (Dr Martin Pölcher) ; Medizinisches Zentrum Bonn - Friedensplatz (Dr Christian Kurbacher) ; Johanniter Krankenhaus, Gynäkologie u. Geburtshilfe, Bonn (Pr Uwe-Jochen Göhring) ; Gemeinschaftspraxis Dr. Hecker und Dr. Lorenz, Braunschweig (Dr Ralf Lorenz) ; Klinikum Bremen-Mitte, Frauenklinik (Pr Willibald Schröder) ; St. Joseph-Stift, Frauenklinik, Bremen (Pr Christiane Frantzen) ; Klinikum Chemnitz gGmbH, Frauenklinik (Dr Petra Krabisch) ; Klinikum Coburg gGmbH, Frauenklinik, (Dr Hermann Zoche) ; Klinikum Darmstadt, Frauenklinik (Dr Sven Ackerman); Klinikum des Landkreises Deggendorf, Frauenklinik (Dr Ronaldo Stuth) ; Städtisches Klinikum, Frauenklinik, Dessau (Dr Inken Achtert) ; St. Johannes Hospital, Frauenheilkunde u. Geburtshilfe, Dortmund (Dr Georg Kunz) ; Universitätsklinikum Carl Gustav Carus, Klinik u. Poliklinik f. Frauenheilkunde u. Geburtshilfe, Dresden (Dr Ulrich Canzler) ; Onkologische Gemeinschaftspraxis, Dresden (Dr Steffen Dörfel) ; Kreisklinik Ebersberg gGmbH, Gynäkologie u. Geburtshilfe (Pr Cornelia Höß) ; Universität Erlangen-Nürnberg, Klinik f. Frauenheilkunde, Erlangen (Pr Matthias W. Beckmann) ; Universitätsklinikum, Frauenklinik, Essen (Dr Pauline Wimberger) ; Elisabeth-Krankenhaus, Klinik f. Gynäkologie u. Geburtshilfe, Essen (Pr Stefan Niesert) ; Klinikum Esslingen, Klinik f. Frauenheilkunde u. Geburtshilfe (Dr Cornelia Kurz) ; Diakonissenanstalt, Frauenklinik, Flensburg (Dr Horst Ostertag) ; Klinikum der J.W. Goethe-Universität, Klinik f. Gynäkologie u. Geburtshilfe, Frankfurt/M. (Dr Lars Hanker) ; Klinikum Frankfurt-Höchst GmbH, Klinik f. Gynäkologie u. Geburtshilfe (Pr Volker Möbus) ; Onkologische Gemeinschaftspraxis, Frankfurt/M. (Pr Hans Tesch) ; Universitäts-Frauenklinik Freiburg (Pr Annette Hasenburg) ; Praxis f. Interdisziplinäre Onkologie u. Hämatologie, Freiburg (Dr Norbert Marschner) ; Kreiskrankenhaus Freudenstadt, Gynäkologie (Andreas Kuznik) ; Klinikum Fürth, Frauenklinik Nathanstift, (Pr Volker Hanf) ; Herz-Jesu-Krankenhaus gGmbH, Frauenheilkunde u. Geburtshilfe, Fulda (Dr Dietrich Mosch) ; Klinikum St. Georg, Franziskus Hospital Harderberg, Gynäkologie, Georgsmarienhütte (Dr Michael Hoedemaker) ; Universitätsklinikum, Zentrum f. Frauenheilkunde u. Geburtshilfe, Gießen (Pr Karsten Münstedt); Kreiskrankenhaus Gifhorn, Gynäkologie (Dr Thomas Dewitz) ; Klinikum Göttingen, Georg-August-Universität, Universitäts-Frauenklinik (Pr Günter Emons) ; Klinikum d. Ernst-Moritz-Arndt-Universität, Klinik u. Poliklinik f. Gynäkologie u. Geburtshilfe, Greifswald (Dr Antje K. Belau) ; Städtisches Klinikum, Frauenklinik, Gütersloh (Dr Joachim Hulde) ; Med. Hochschule Hannover, Frauenklinik (Pr Tjoung-Won Park-Simon) ; Universitätsklinikum Hamburg-Eppendorf, Klinik u. Poliklinik f. Frauenheilkunde u. Geburtshilfe, Hamburg (Dr Sven Mahner) ; Albertinen Krankenhaus, Gynäkologie, Hamburg (Dr Uwe Herwig) ; Asklepios Klinik Nord, Campus Heidberg, Innere Medizin, Hamburg (Dr Peter Müller) ; Klinikum Hanau gGmbH, Frauenklinik (Dr Thomas Müller) ; Henriettenstiftung, Frauenklinik, Hannover (Dr Iris Schrader) ; Ev. Diakoniewerk Friederikenstift, Frauenklinik, Hannover (Dr Jörg Gade) ; Paracelsus-Klinik, Frauenklinik, Henstedt-Ulzburg (Dr Barbara Jahns); Gemeinschaftspraxis f. Gynäkologie, Hildesheim (Dr Christoph Uleer); Klinikum Itzehoe, Klinik f. Frauenheilkunde u. Geburtshilfe (Dr Britta Seifert); Universitätsklinikum, Klinik f. Frauenheilkunde u. Geburtshilfe, Jena (Pr Ingo Runnebaum); St. Vincentius Kliniken gAG, Frauenklinik, Karlsruhe (Dr Anne Stähle); Städtisches Klinikum, Frauenklinik, Karlsruhe (Pr Hans-Ulrich Ulmer); Diakonissenkrankenhaus Karlsruhe, Frauenklinik, Karlsruhe (Dr Gerhard Deutsch); Klinikum Kassel, Frauenklinik (Dr Hans Urbanczyk); Universitätsklinikum Schleswig-Holstein, Campus Kiel, Klinik f. Gynäkologie u. Geburtshilfe (Dr Felix Hilpert); Klinikum der Universität zu Köln, Klinik u. Poliklinik f. Frauenheilkunde u. Geburtshilfe (Dr Joachim Schneider); Ev. Krankenhaus Köln-Kalk GmbH, Gynäkologie u. Geburtshilfe (Dr Wolfgang Maurer); Klinikum Lahr, Frauenklinik (Dr Daniel Pfisterer); Praxis Vehling-Kaiser Hämatologie/Onkologie, Landshut

(Dr Ursula Vehling-Kaiser); Kreiskrankenhaus Leonberg, Frauenklinik (Dr Harald Wolf); St. Vincenz Krankenhaus, Frauenklinik, Limburg (Dr Peter Scheler); St. Elisabethen-Krankenhaus gGmbH, Zentrum Gynäkologie u. Geburtshilfe, Lörrach (Dr Brigitte Schuhmann); Universitätsklinikum Schleswig-Holstein, Campus Lübeck, Klinik f. Frauenheilkunde u. Geburtshilfe (Dr Marc Thill); Städtisches Klinikum, Frauenklinik, Lüneburg (Dr Eric Boetel); Kath. Klinikum, St. Vincenz u. Elisabeth Krankenhaus, Frauenklinik, Mainz (Pr Wolfgang Wiest); Otto-von-Guericke-Universität, Klinik f. Frauenheilkunde u. Geburtshilfe, Magdeburg (Dr Joachim Bischoff); Städtisches Klinikum, Standort Olvenstedt, Klinik f. Hämatologie/Onkologie, Magdeburg (Dr Erika Kettner); Klinikum d. Johannes Gutenberg Universität, Klinik u. Poliklinik f. Geburtshilfe u. Frauenheilkunde, Mainz (Dr Marcus Schmidt); Klinikum Mannheim gGmbH, Universitätsklinikum, Frauenklinik (Pr Marc Sütterlin); Universitätsklinikum Gießen u. Marburg GmbH, Klinik f. Gynäkologie, Gynäkologische Endokrinologie u. Onkologie , Marburg (Dr Klaus Baumann); Klinikum Großhadern, Ludwig-Maximilians-Universität, Frauenklinik, München (Dr Alexander Burges); Klinikum Innenstadt, Ludwig-Maximilians-Universität, Klinik u. Poliklinik f. Frauenheilkunde u. Geburtshilfe, München (Pr Harald Sommer); Städtisches Klinikum Neunkirchen gGmbH, Frauenklinik (Dr Georg-Peter Breitbach); Dietrich-Bonhoeffer-Klinikum, Klink f. Frauenheilkunde u. Geburtshilfe, Neubrandenburg (Katrin Borkowski); Städtisches Kliniken Neuss, Lukaskrankenhaus GmbH, Frauenklinik (Pr Hans-Georg Schnürch); Marienhaus Klinikum, St. Elisabeth Krankenhaus, Klinik f. Gynäkologie u. Geburtshilfe, Neuwied (Pr Richard Berger); Klinikum Offenbach, Klinik f. Gynäkologie u. Geburtshilfe (Dr Jens Kosse); Ortenau Klinikum, St. Josefsklinik, Gynäkologie, Offenburg (Dr Dietmar Schwörer); Asklepios Klinik, Gynäkologie u. Geburtshilfe, Bad Oldesloe (Dr Tobias Zeiser); Klinikum "Ernst-von-Bergmann", Gynäkologie, Potsdam (Dr Friedrich Dreßler); Klinikum „Dorothea Christiane Erxleben", Frauenklinik, Quedlinburg (Dr Sven-Thomas Graßhoff); Elblandkliniken Meißen-Radebeul GmbH & Co. KG, Frauenklinik, Radebeul (Dr Barbara Richter); Oberschwaben Klinik, Krankenhaus St. Elisabeth, Studienzentrum, Ravensburg (Dr Gerhard Fischer); Klinikum am Steinenberg, Frauenklinik, Reutlingen (Peter Krieger); Klinikum Südstadt der Hansestadt Rostock, Universitäts-Frauenklinik u. Poliklinik (Pr Bernd Gerber); Klinikum Rosenheim, Klinik f. Gynäkologie u. Geburtshilfe, Rosenheim (Pr Thomas Beck); Kreiskrankenhaus Rottweil, Frauenklinik (Dr Gerhard Bartzke); Gemeinschaftspraxis f. Frauenheilkunde u. Geburtshilfe, Salzgitter (Dr Wolfgang Dietz); Krankenhaus am Rosarium GmbH, Klinik f. Frauenheilkunde u. Geburtshilfe, Sangerhausen (Dr Cornelia Jacob); Leopoldina-Krankenhaus GmbH, Frauenklinik, Schweinfurt (Pr Michael Weigel); St. Marien-Krankenhaus, Frauenklinik, Siegen (Dr Matthias Stamm); Ev. Jung-Stilling-Krankenhaus, Frauenklinik, Siegen (Dr Volker Müller); Kreiskrankenhaus Sigmaringen, Gynäkologie (Dr Peter Krezdorn); Klinikum Schaumburg, Kreiskrankenhaus Stadthagen, Frauenklinik (Dr Johannes Feltz-Süßenbach); Robert-Bosch-Krankenhaus GmbH, Hämatologie/Onkologie, Stuttgart (Pr Walter Aulitzky); Marienhospital, Gynäkologie, Stuttgart (Dr Heinrich Stehle); Kreiskrankenhaus Torgau „Johann Kentmann" gGmbH, Gynäkologie u. Geburtshilfe (Dr Eike Simon); Hämatologisch-Onkologische Schwerpunktpraxis, Troisdorf (Dr Helmut Forstbauer); Universitäts-Frauenklinik Tübingen (Pr Erich Solomayer); Universitäts-Frauenklinik, Ulm (Pr Rolf Kreienberg); Kreiskrankenhaus Waiblingen, Klinik f. Gynäkologie u. Geburtshilfe (Edina Sulea) Marien-Hospital Witten gGmbH, Frauenklinik (Dr Felix Bartzsch);
HSK, Dr. Horst Schmidt Klinik, Klinik f. Gynäkologie u. Gynäkologische Onkologie, Wiesbaden (Dr Philipp Harter); St. Josefs-Hospital, Gynäkologie u. Geburtshilfe, Wiesbaden (Pr Gerald Hoffmann); Klinikum Worms gGmbH, Frauenklinik (Pr Thomas Hitschold); Klinikum der Stadt Wolfsburg, Frauenklinik (Pr Karl Ulrich Petry); Praxis f. Hämatologie u. internistische Onkologie. Wuppertal (Dr Werner Fett); Missionsärztliche Klinik Würzburg gGmbH, Gynäkologie u. Geburtshilfe (Pr Dietmar Kranzfelder).

ANZGOG group: Philip Beale, Julie Martyn, Kim Gillies
Australia: Border Medical Oncology (Dr Christopher Steer); Box Hill Hospital (Dr Geraldine Goss); Mercy Hospital for Women (Pr Danny Rischin); Calvary Mater Newcastle Hospital (Dr Tony Bonaventura); Prince of Wales Hospital (Pr Michael Friedlander); Royal Adelaide Hospital (Dr Margaret Davy); Royal Brisbane Hospital (Dr Jeffrey Goh); Royal Hobart Hospital (Dr Rob Mc Intosh); Royal North Shore Hospital (Dr Sally Baron-Hay); Royal Prince Alfred Hospital (Dr Philip Beale); Royal Women s Hospital (Pr Michael Quinn); Sir Charles Gairdner Hospital (Dr Martin BUCK); Wesley Medical Centre (Dr Paul Vasey); Lismore Base Hospital (Dr Adam Boyce); Canberra Hospital (Dr Alison Davis); Cabrini Institute (Dr Gary Richardson).
New Zealand: Christchurch Hospital (Dr Bernie Fitzharris); Dunedin Hospital (Dr David Perez); Wellington Hospital (Dr Anne O Donnell)

GINECO group: Eric Pujade Lauraine, Frédéric Marmion, Benedicte Votan
Centre Catherine de Sienne, Nantes (Dr Alain Lorholary); Institut Bergonié, Bordeaux (Dr Anne Floquet); Centre François Baclesse, Caen (Pr Florence Joly); Centre Alexis Vautrin-Brabois, Vandoeuvre-les-nancy (Dr Béatrice Weber); Hôpital Hôtel-Dieu, Paris (Pr Eric Pujade–Lauraine); Clinique Armoricaine de Radiologie, Saint-Brieuc (Dr Anne-Claire Hardy-Bessard); Hôpital Cochin, Paris (Pr François Goldwasser); Hôpital Européen Georges Pompidou, Paris (Dr Eric Levy); Centre Paul Papin, Angers (Dr Rémy Delva); Hôpitaux Civils de Colmar, Colmar (Dr Jean-Claude Barats); CRLC Val d'Aurelle, Montpellier (Dr Michel Fabbro); Institut Curie, Paris (Dr Paul-Henri Cottu); Centre d'Oncologie de Gentilly, Nancy (Dr Dominique Spaeth); Centre Léon Bérard, Lyon (Dr Isabelle Ray-Coquard); Centre René Huguenin, Saint-Cloud (Dr Fawzia Mefti); Hôpital Civil, Strasbourg (Pr Jean-Emmanuel Kurtz); Hôpital Tenon, Paris (Dr Frédéric Selle); Institut Sainte-Catherine, Avignon (Dr Gaëtan de Rauglaudre); Centre René Gauducheau, Saint-Herblain (Dr Dominique Berton-Rigaud); Hôpital André Mignot, Le Chesnay (Dr Jean-François Geay); Centre Henri Becquerel, Rouen (Dr Cécile Guillemet); Clinique Jean Bernard, Le Mans (Dr Hugues Bourgeois); Centre Antoine Lacassagne, Nice (Dr Véronique Mari); CHD Les Oudairies, La Roche-sur-Yon (Dr Franck Priou); Centre Claudius Regaud, Toulouse (Dr Laurence Gladieff); Centre Jean Perrin, Clermont-Ferrand (Dr Xavier Durando); Hôpital Saint-Louis, Paris (Dr Jean-Louis Misset); Institut Gustave Roussy, Villejuif (Dr Catherine Lhomme); Centre Oscar Lambret, Lille (Dr Anne Lesoin); Centre Hospitalier des Diaconesses, Paris (Dr Anne Plantade); Hôpital Mont-de-Marsan, Mont-de-Marsan (Dr Jérôme Dauba) CHRU Lille - Hôpital Huriez, Lille (Dr Mohammed Hebbar); Hôpital Foch, Suresnes (Dr Mehran Khatibi); Institut Jean Godinot, Reims (Pr Hervé Cure)

GEICO group: Andres Cervantes, Federico Nepote, Andres Poveda
Hospital Clínico Universitario de Valencia (Dr Andrés Cervantes); Instituto Valenciano de Oncología (Dr Andrés Poveda); Hospital Universitario La Fe, Valencia (Dr Ana Santaballa); Hospital Ramón y Cajal, Madrid (Dr Eva Mª Guerra) ; Hospital Valle Hebrón, Barcelona (Dr José María Del Campo) ; Durán y Reynals, Barcelona (Dr Beatriz Pardo Burdalo); Hospital Sant Pau, Barcelona (Dr Belén Ojeda); Hospital Clinic Barcelona, Barcelona (Dr Pere Gascón); Hospital Son Llatzer, Mallorca (Dr Isabel Bover); Hospital Marqués de Valdecilla, Santander (Dr Ana De Juan); Hospital Miguel Servet, Zaragoza (Dr Ana Herrero); Hospital Josep Trueta, Girona (Dr Miguel Beltrán); Hospital Universitario La Paz, Madrid (Dr Andres Redondo).

MRC/NCRI group: Timothy J Perren, Ann Marie Swart, Cybil Kwakye, Laura Farrelly, Wendi Qian, Mahesh Parmar, Rick Kaplan, Adrian Cook, Andrew Embleton
St James University Hospital, Leeds (Dr Timothy Perren); Christie Hospital, Manchester (Dr Jurjees Hasan); Clatterbridge Centre for Oncology, Wiral (Dr John Green); Derriford Hospital, Plymouth (Dr Dennis Yiannakis); Mount Vernon Hospital, Northwood (Pr Gordon Rustin); Queen Elizabeth Hospital, Gateshead (Dr Raj Naik); Queen Elizabeth The Queen Mother Hospital, Margate (Dr Justin Waters); Queen Alexandra Hospital, Portsmouth (Dr Yoodhivir Nagar); University College London Hospital, London (Pr Jonathan Ledermann); Weston Park Hospital, Sheffield (Dr Simon Pledge); Maidstone Hospital, Maidstone (Dr Jeff Summers); Cancer Research UK, London (Pr Iain McNeish); Southampton General Hospital, Southampton (Dr Sajid Durrani); Royal Surrey County Hospital, Guildford (Dr Sharadah Essapen); Wexham Park Hospital, Slough (Dr Marcia Hall); Churchill Hospital, Oxford (Pr Bass Hassan); Castle Hill Hospital, Cottingham (Dr Penelope O Neill); Queens Hospital, Burton-on-Trent, (Dr Mojca Persic); St Georges Hospital, London (Dr Fiona Lofts); Freeman Hospital, Newcastle upon Tyne (Dr Graham Dark); Yeovil District Hospital, Yeovil (Dr Geoffrey Sparrow); New Cross Hospital, Wolverhampton (Dr Rozenn Allerton); Ninewells Hospital, Dundee (Dr Michelle Ferguson); The Ipswich Hospital, Ipswich (Dr Jamey Morgan); Nottingham City Hospital, Nottingham (Dr Steven Chan); Cheltenham General Hospital, Cheltenham (Dr Radi Counsell); Royal Derby Hospital, Derby (Dr Mojca Persic); University Hospital Coventry and Warwickshire NHS Trust, Coventry (Dr Clive Irwin); Royal Marsden Hospital, London (Pr Martin Gore); Royal Marsden, Sutton, (Pr Martin Gore); Broomfield Hospital, Chelmsford (Dr Saad Tahir); Hammersmith Hospital, London (Dr Sarah Blagden); James Cook University Hospital, Middlesbrough (Dr Sath Gokul); Poole Hospital, Poole (Dr Richard Osbourne); North Devon District Hospital, Barnstaple (Dr Mark Napier); Great Western Hospital, Swindon (Dr Amanda Horne); Royal Cornwall Hospital, Truro, (Dr Nigel Bailey); Aberdeen Royal Infirmary, Aberdeen (Dr Radha Todd); Guys Hospital, London (Dr Peter Harper); Huddersfield Royal Infirmary, Huddersfield (Dr Barbara Crosse); Addenbrookes Hospital, Cambridge (Dr Helena Earl); City Hospital, Birmingham (Dr Ahmed El-Modir); Gartnavel General Hospital, Glasgow (Dr Nick Reed); Leicester Royal Infirmary, Leicester (Dr Paul Symonds); Velindre Hospital, Cardiff (Dr Malcolm Adams); Queens Hospital, Romford, (Dr Mary Quigley); Essex County Hospital, Colchester (Dr Alan Lamont).

NSGO group: Gunnar Kristensen, Mansoor Raza Mirza
Denmark: Aalborg University Hospital (Dr Bente Lund); Odense University Hospital (Dr Mansoor Raza Mirza; Pr Jørn Herrstedt); Herlev University Hospital (Pr Jørn Herrstedt ,Dr Hanne Havsteen); Regionhospitalet, Herning (Dr Nina Keldsen); Rigshospitalet, Copenhagen University Hospital (Dr Svend Aage Engelholm).
Finland: Oulu University Hospital (Dr Ulla Puistola); Turku University Hospital (Dr Seija Grenman); Helsinki University Central Hospital (Dr Arto Leminen).
Sweden: Karolinska University Hospital, Stockholm (Dr Bengt Tholander); Lindköping University Hospital (Dr Per Rosenberg); Örebro University Hospital (Pr Bengt Sorbe); Lund University Hospital (Dr Kjell Bergfeldt).
Norway: The Norwegian Radium Hospital Oslo (Dr Gunnar Kristensen); Haukeland University Hospital, Bergen (Dr Harald Helland); St Olavs Hospital Trondheim (Dr Ingrid Baasland); Stavanger University Hospital (Dr Bent Fiane).

NCIC Clinical trials Group: Ralph Meyer, Cathy Davidson, Monica Bacon, Brenda Evans, Mark Carey
Dr. H. Bliss Murphy Cancer Centre, NL (Dr Patti Power); QEII Centre for Clinical Research, Halifax (Dr Robert Grimshaw); The Moncton Hospital, Moncton (Dr Sheldon Rubin); Centre Hospitalier Universitaire de Sherbrooke, Sherbrooke (Dr Paul Bessette); CHUQ - Hotel-Dieu de Quebec, Quebec (Dr Marie Plante); McGill University-Jewish General Hospital, Montreal (Dr Walter Gotlieb); Hopital Notre-Dame, Montreal (Dr Diane Provencher); Juravinski Cancer Centre, Hamilton (Dr Laurie Elit); Univ. Health Network/Princess Margaret Hospital, Toronto (Dr Amit Oza); Credit Valley Hospital, Mississauga (Dr Leonard Kaizer); London Regional Cancer Centre, London (Dr Monique Bertrand); Algoma District Program Sault Area Hospital, Ontario (Dr Silvana Spadafora); Cancer Care Manitoba, Winnipeg (Dr Robert Lotocki); Allan Blair Cancer Centre, Regina (Dr Haji Chalchal); Tom Baker Cancer Centre, Calgary (Dr Prafull Ghatage); Cross Cancer Institute, Edmonton (Dr Valerie Capstick); BCCA-Vancouver (Dr Paul Hoskins); BCCA-Fraser Valley (Dr Ursula Lee); BCCA-Kelowna (Dr Susan Ellard); Vancouver Coastal Health (Dr. Mark Carey).

Trial Management Group: T Perren, A Oza, AM Swart, A Cook, A Embleton, L Farrelly, R Kaplan, E Kent, C Kwakye, M Parmar, W Qian, L Morales, P Beale, M Friedlander, K Gillies, J Martyn, J Simard-Lebrun, K Scott, M Bacon, M Carey, C Davidson, E Eisenhauer, D Tu, F Marmion, E Pujade-Lauraine, G Elser, A Krueger, J Pfisterer, N Colombo, R Fossati, T Kirkegaard, G Kristensen, M Raza, A Cervantes, G Jayson, D Stark, D Epstein, S Hinde, M Sculpher
Trial Physicians: F Collinson, S Sim, F Al-Terkait
Independent Data monitoring Committee: R Coleman, W Sauerbrei, U Menon, R Buckstein
International Trial Steering Committee: C Parker, R Rudd, J Whelan, D Fink, M Mason, P Johnson

Supplementary Table 1. OS in high-risk patients: Current, previous ICON7 and GOG-218 high-risk definitions.

	Current definition		Previous ICON7		GOG-0218	
	**Standard therapy**	**Bevacizumab**	**Standard therapy**	**Bevacizumab**	**Standard therapy**	**Bevacizumab**
**patients (n)**	254	248	237	235	377	396
**mths follow-up, median (IQR)**	28.8 (14.0,50.5)	38.7 (21.0,52.3)	29.9 (16.0,51.4)	38.6 (21.3,52.5)	34.9 (19.3,52.3)	39.6 (23.4,53.3)
**events n(%)**	174 (69%)	158 (64%)	159 (67%)	150 (64%)	246 (65%)	248 (63%)
**median survival (mths)**	30.1	39.5	31.2	39.3	36.9	40.9
**log-rank**	p=0.03		p=0.07		p=0.10	
**HR (95% CI)**	0.78 (0.63,0.97)		0.81 (0.65, 1.02)		0.86 (0.72,1.03)	
**non-proportionality**†	p=0.01		p=0.003		p=0.20	
**(restricted) mean survival time**††	34.4	39.2	35.0	39.6	37.7	40.5
**RMST difference (95%CI)**	4.8 (1.3,8.3)		4.6 (1.0,8.1)		2.8 (0.3,5.2)	

† Grambsch-Therneau test
†† restricted at 5 years

Supplementary Table 2. Baseline characteristics.

	All		High risk	
	**Standard therapy**	**Bevacizumab**	**Standard therapy**	**Bevacizumab**
	**(n=764)**	**(n=764)**	**(n=254)**	**(n=248)**
**Age, median(range)**				
**Years**	57 (18-81)	57 (24-80)	60 (18,81)	60 (26,80)
**Race, n(%)**				
**White**	737 (96)	730 (96)	242 (95)	237 (96)
**Asian/Black/Other**	27 (4)	34 (4)	12 (5)	11 (4)
**ECOG PS, n(%)**				
**0**	360 (48)	335 (45)	98 (39)	100 (41)
**1**	354 (47)	366 (49)	135 (53)	123 (50)
**2**	41 (5)	45 (6)	20 (8)	21 (9)
**unknown**	9	18	1	4
**Origin of cancer, n(%)**				
**Ovary (epithelial)**	667 (87)	673 (88)	210 (83)	207 (83)
**Fallopian tube**	29 (4)	27 (4)	5 (2)	6 (2)
**Primary peritoneal**	56 (7)	50 (7)	32 (13)	28 (11)
**Multiple sites**	12 (2)	14 (2)	7 (3)	7 (3)
**Histology, n(%)**				
**Serous**	529 (69)	525 (69)	195 (77)	186 (75)
**Mucinous**	15 (2)	19 (2)	4 (2)	6 (2)
**Endometroid**	57 (7)	60 (8)	14 (6)	17 (7)
**Clear cell**	60 (8)	67 (9)	6 (2)	6 (2)
**Mixed**	48 (6)	40 (5)	14 (6)	14 (6)
**Other**	55 (7)	53 (7)	21 (8)	19 (8)
**FIGO stage, n(%)**				
**I/IIA**	75 (10)	67 (9)		
**IIB/IIC**	70 (9)	70 (9)		
**III**	14 (2)	18 (2)	6 (2)	6 (2)
**IIIA**	32 (4)	22 (3)	2 (0.8)	2 (0.8)
**IIIB**	44 (6)	45 (6)	8 (3)	6 (2)
**IIIC**	432 (57)	438 (57)	141 (56)	130 (52)
**IV**	97 (13)	104 (14)	97 (38)	104 (42)
**Grade, n(%)**				
**Grade 1**	56 (7)	41 (5)	10 (4)	5 (2)
**Grade 2**	142 (19)	175 (23)	45 (18)	70 (29)
**Grade 3**	556 (74)	538 (71)	195 (78)	169 (69)
**unknown**	10	10	4	4
**Debulking surgery**				
**Inoperable**	17 (2)	13 (2)	17 (7)	13 (5)
**>1cm residual**	199 (26)	196 (26)	194 (76)	194 (78)
**0-1cm residual**	175 (23)	194 (25)	19 (7)	20 (8)
**0cm residual***	373 (49)	361 (47)	24 (9)	21 (8)

* includes 20 ref and 21 bev pts with residual ≤1cm, exact size unknown (high risk, 2 ref and 2 bev)

Supplementary Table 3. Baseline characteristics of pre-specified patient subgroups.

	Clear cell		Low stage high grade		Low grade serous	
	**Standard therapy**	**Bevacizumab**	**Standard therapy**	**Bevacizumab**	**Standard therapy**	**Bevacizumab**
**patients (n)**	77	82	75	67	49	31
**Age, median(range) years**	56 (29-75)	54 (25-75)	54 (29-75)	54 (40-72)	49 (24-71)	47 (24-79)
**ECOG PS, n(%)**						
**0**	42 (56)	39 (49)	46 (63)	37 (57)	20 (41)	16 (55)
**1**	32 (43)	37 (46)	27 (37)	28 (43)	27 (55)	12 (41)
**2**	1 (1)	4 (5)	0 (0)	0 (0)	2 (4)	1 (3)
**unknown**	2	2	2	2	0	2
**FIGO stage, n(%)**						
**I/IIA**	37 (48)	30 (37)	75 (100)	67 (100)	0 (0)	0 (0)
**IIB/IIC**	8 (10)	16 (20)			4 (8)	2 (6)
**III**	0 (0)	2 (2)			0 (0)	1 (3)
**IIIA**	2 (3)	2 (2)			7 (14)	2 (6)
**IIIB**	3 (4)	3 (4)			5 (10)	3 (10)
**IIIC**	23 (30)	24 (29)			33 (67)	20 (65)
**IV**	4 (5)	5 (6)			0 (0)	3 (10)
**Grade, n(%)**						
**Grade 1**	1 (1)	1 (1)	0 (0)	0 (0)	49 (100)	31 (100)
**Grade 2**	4 (5)	2 (2)	1 (1)	1 (1)		
**Grade 3**	72 (94)	79 (96)	74 (99)	66 (99)		

Supplementary Table 4. Global Quality of Life at week 76.

	Reference (N=764)			Bevacizumab (N=764)				
	**mean**	**(sd)**	**n**	**mean**	**(sd)**	**n**	**p[a]**	**Bev-Ref (95%CI)**
**Baseline[b]**								
**All**	57.0	(20.0)	745	54.7	(19.1)	740		
**Non high risk**	58.5	(19.9)	495	56.3	(18.6)	497		
**High risk**	54.1	(20.1)	250	51.5	(19.8)	243		
**Week 76**								
**All**	75.9	(19.3)	175	72.6	(18.9)	199	0.43	-1.47 (-5.11,2.18)
**Non high risk**	76.5	(19.2)	149	71.5	(19.1)	155	0.02	-5.1 (-9.4,-0.7)
**High risk**	72.4	(19.4)	26	76.7	(18.0)	44	0.36	4.3 (-4.9,13.4)
**Sensitivity analysis[c]**								
**Non high risk**	64.4	(17.3)	320	61.3	(15.1)	368	0.01	-3.1 (-5.6,-0.71)
**High risk**	61.4	(14.2)	72	63.5	(15.3)	114	0.36	2.1 (-2.4,6.5)

a. adjusted for baseline score
b. screening and baseline scores averaged for each patient
c. imputed score of mean-20 points for patients missing but not known died or progressed; lower score expected in missing patients if some are missing due to illness

Supplementary Figure 1. High-risk group composition

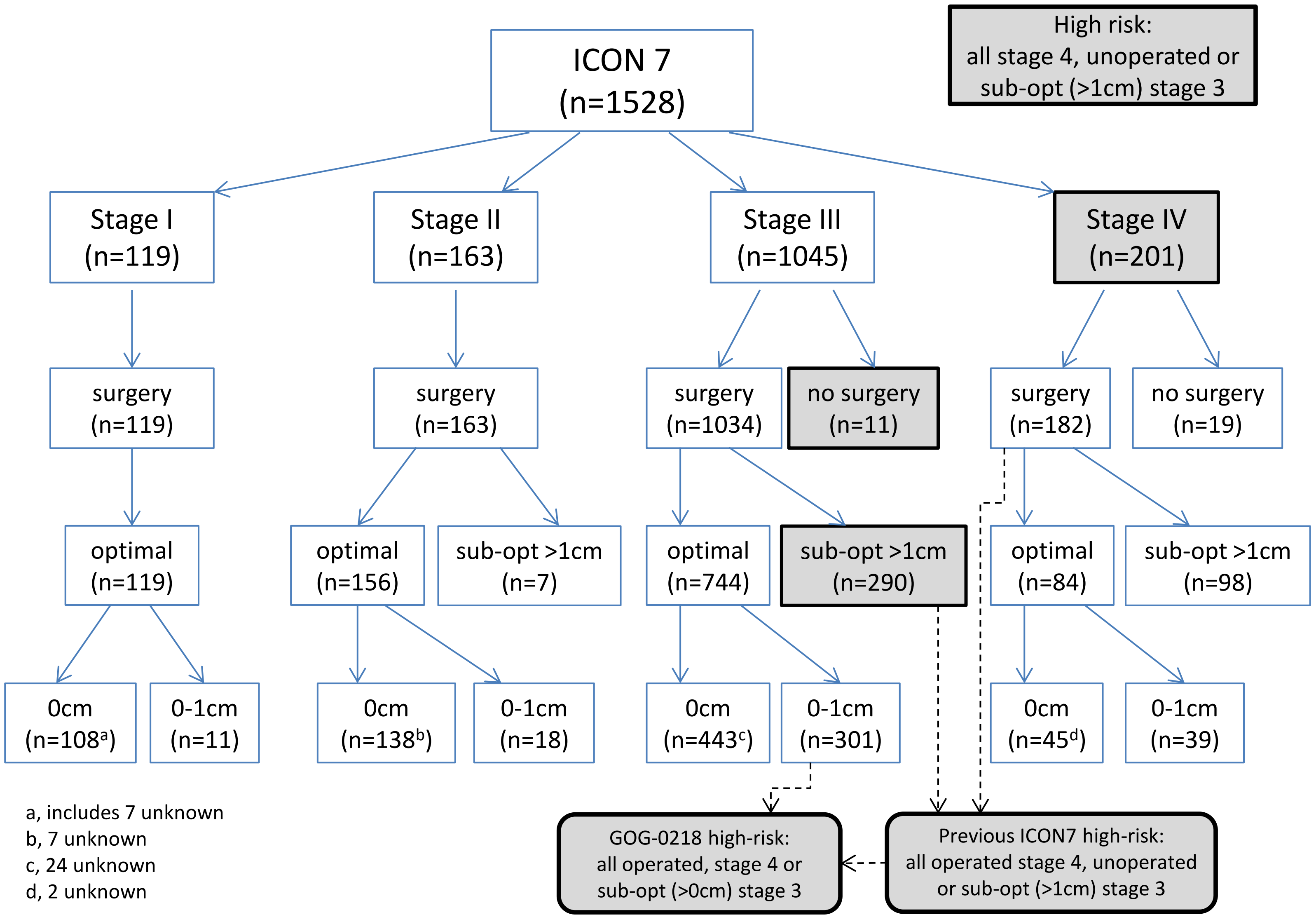

Supplementary Figure 2. Updated PFS, all patients and high-risk subgroup

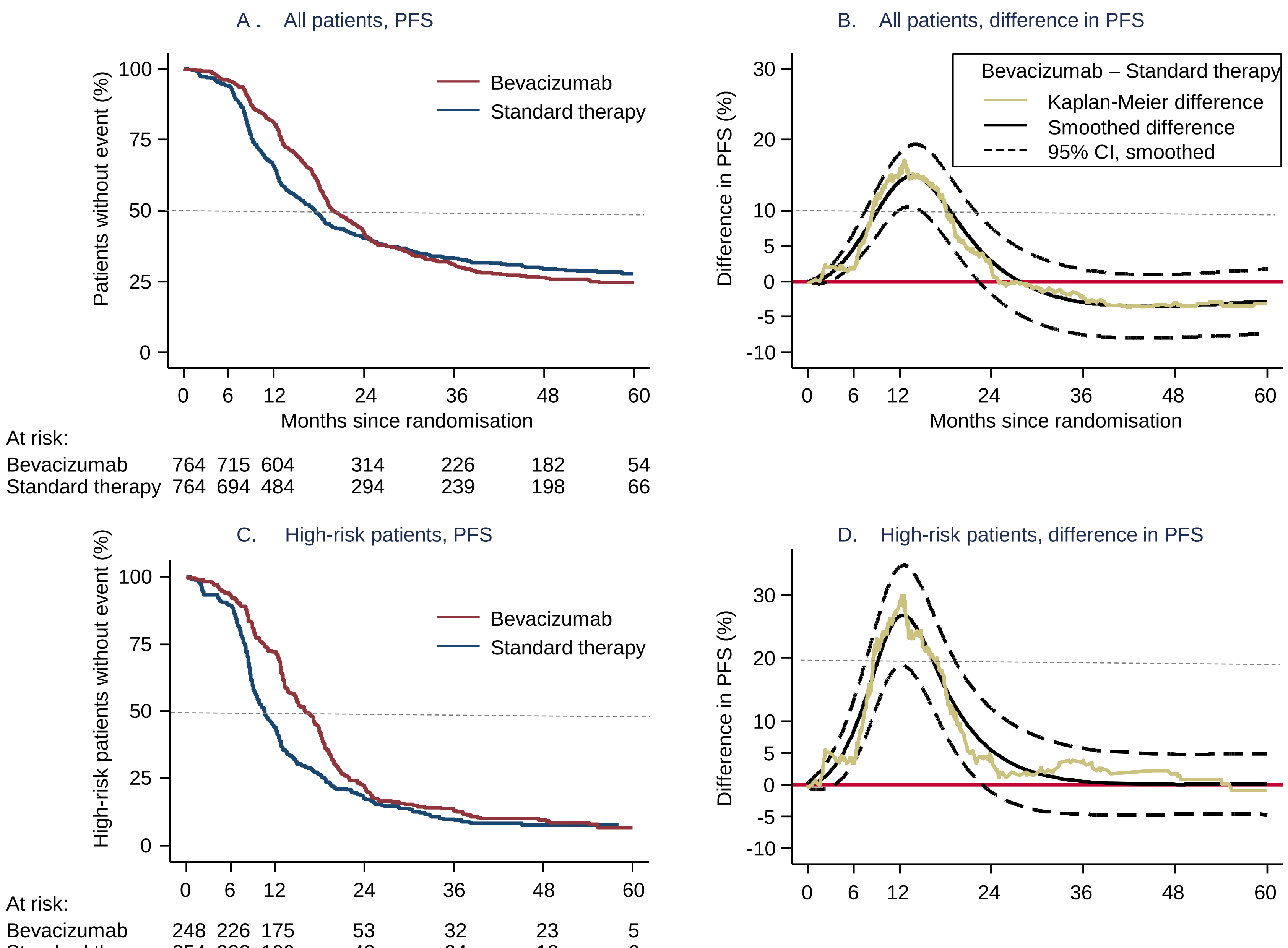

Supplementary Figure 3. Treatment effect on PFS disease status at enrolment

	events/n	hazard ratio (95% CI)	mean survival, months (95%CI) bevacizumab	control	
All patients	1080/1528	0.93 (0.83,1.05)	29.2 (27.7,30.7)	27.6 (26.1,29.2)	
Non high-risk	629/1026	1.03 (0.88,1.21)	33.7 (31.9,35.5)	33.8 (31.8,35.7)	Interaction p=0.005
High risk	451/502	0.73 (0.61,0.88)	20.0 (18.1,21.8)	15.9 (14.1,17.7)	
Stage I,II,III (0cm)	378/725	0.97 (0.79,1.18)	39.2 (37.1,41.4)	37.9 (35.7,40.2)	
Stage III (>0, ≤1cm)	251/301	1.07 (0.83,1.38)	21.3 (19.0,23.7)	22.7 (19.5,25.9)	
Stage III (>1cm)	260/290	0.75 (0.59,0.96)	20.8 (18.3,23.2)	17.0 (14.6,19.3)	Trend p=0.014
Stage IV	163/182	0.75 (0.55,1.02)	19.3 (16.4,22.3)	15.5 (12.3,18.7)	
Inoperable	28/30	0.58 (0.26,1.29)	15.8 (7.4,24.2)	10.4 (7.1,13.7)	

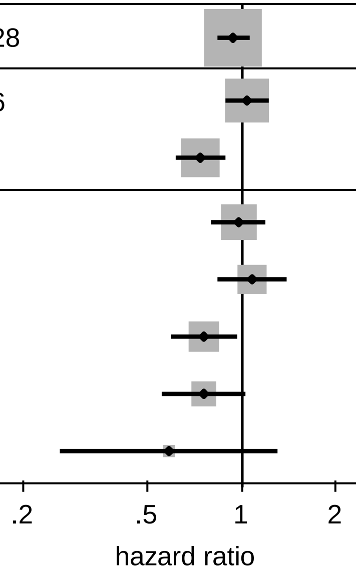

Supplement: Supplementary appendix [file mmc1.pdf]
